# Supplementary material for: Enhanced retinal pigment epithelial cells as a delivery vehicle for retinal disease
Source: Mol Ther Methods Clin Dev. 2025 Mar 14;33(2):101450. doi: 10.1016/j.omtm.2025.101450 (PMC11995081; doi:10.1016/j.omtm.2025.101450)
Supplement: Document S1. Figures S1–S4 [file mmc1.pdf]

OMTM, Volume *33*

Supplemental information

Enhanced retinal pigment epithelial cells as a delivery vehicle for retinal disease

Avril Reddy, Chris Greene, Yosuke Hashimoto, Anna-Sophia Kiang, Natalie Hudson, Peter Adamson, Tiago Santos-Ferreira, and Matthew Campbell

Supplemental Figures

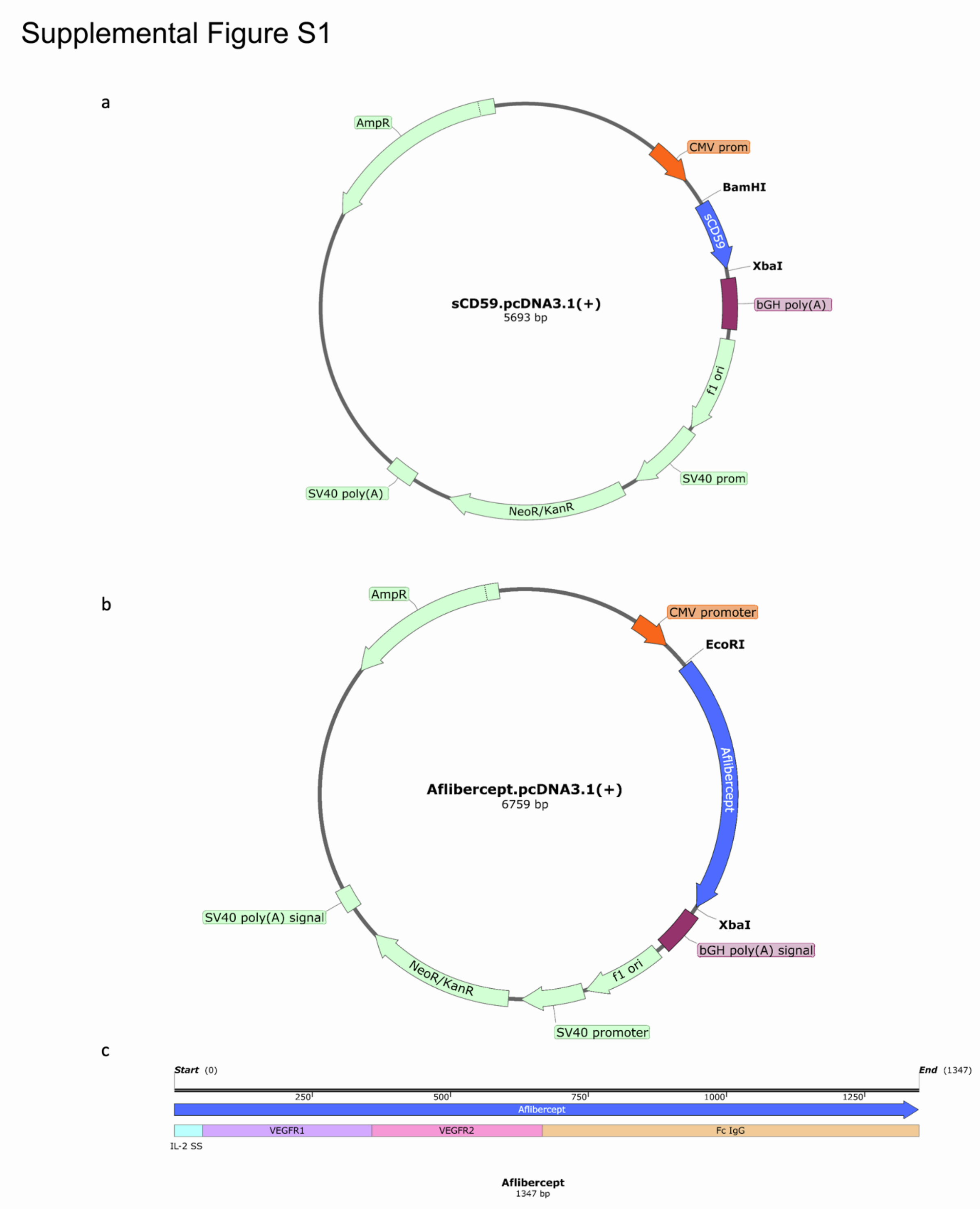

Figure S1. Graphic maps of plasmids used for ARPE-19 transfection.

a) Maps of the sCD59 and **b)** aflibercept expressing plasmids containing the target cDNA (blue) that was confirmed to share sequence identity with published sequences (Homo Sapiens CD59, transcript variant 1, (NM_203330.2) and Drugbank Aflibercept sequence (DB08885),

respectively). Target cDNA is flanked by the BamHI or EcoRI and Xba1 restriction enzyme recognition sites. The ampicillin resistance region (AmpR, green), was utilised for selection of a transformant colony prior to large scale maxiprep. **c)** The aflibercept cDNA sequence contains the interleukein-2 secretion signal peptide (IL-2 SS), vascular endothelial growth factor receptor (VEGFR) 1, VEGFR2 and Fc fragment of human IgG1 immunoglobulin (Fc IgG) sequences.

Supplemental Figure S2

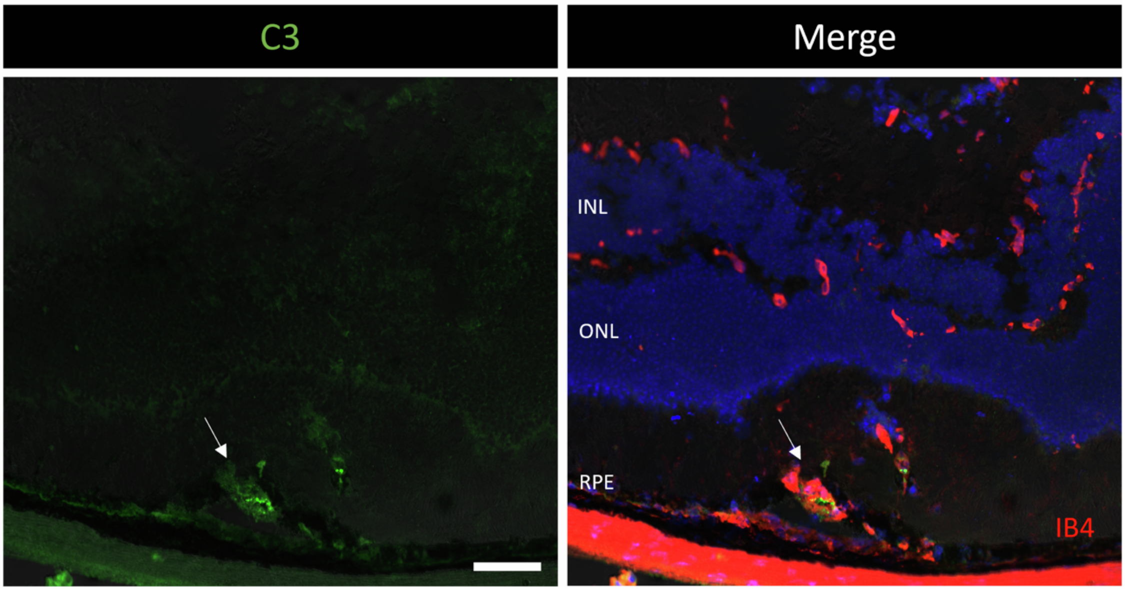

Figure S2. Complement, C3, is observed in areas of neovascular lesions in JR5558 mice. The JR5558 mouse model of choroidal neovascularisation (CNV) shows signs of complement activation such as C3 deposition (green, white arrows) around abnormal blood vessels (red, isolectin-B_4, IB4) in the outer retina. Nuclei marked by Hoechst 33258. INL: inner nuclear layer, ONL: outer nuclear layer, RPE: retinal pigment epithelium. Scale bar = 50µm.

Supplemental Figure S3

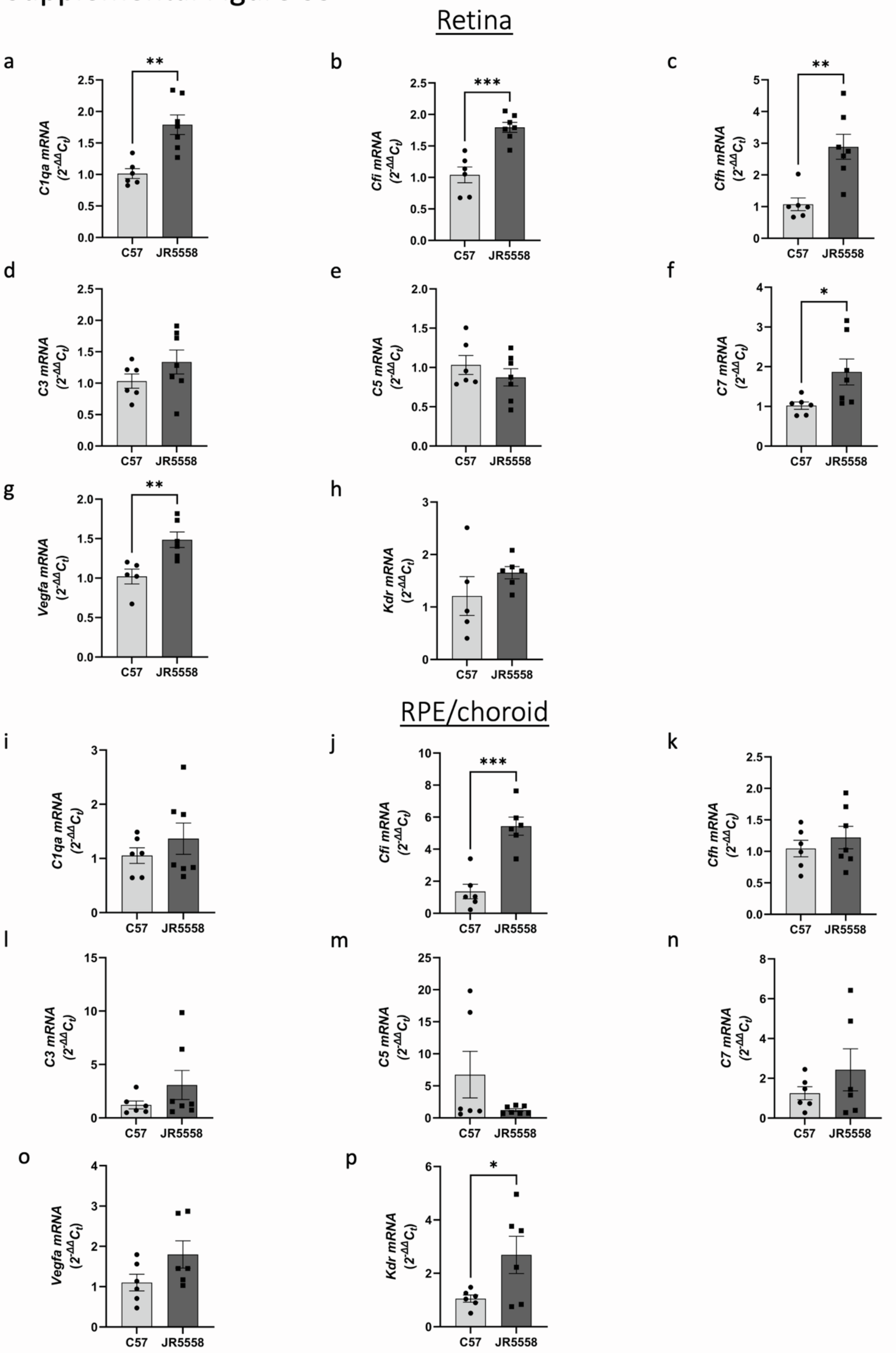

Figure S3. Transcript expression of several complement components and members of the Vegf pathway are upregulated in the retina and RPE/choroid of JR5558 mice.

Signs of complement activation were noted in the retinas of 8-week-old JR5558 mice compared to age-matched C57BL/6J mice. Increased expression of components (a) *C1qa* (**p = 0.0014), (b) *Cfi* (***p = 0.0003), (c) *Cfh* (**p = 0.0025) and (f) *C7* (*p = 0.0401) in the retina and (j) *Cfi* (***p = 0.0002) in the RPE/choroid indicate that the complement system may be activated in this spontaneous neovascular model. Expression of (d) *C3* (p = 0.2142), and (e) *C5* (p = 0.3536) in the retina and (i) *C1qa* (p = 0.3787), (k) *Cfh* (p = 0.4578), (l) *C3* (p = 0.2461), (m) *C5* (p = 0.1452) and (n) *C7* (p = 0.3109) in the RPE/choroid are not significantly different in JR5558 mice. (g) Transcript expression of *Vegfa* is significantly increased in the retina (**p = 0.0082), (o) but not in the RPE/choroid (p = 0.1082) of JR5558 mice. (h) Conversely, *Kdr,* (the murine homolog of VEGFR2) is unchanged in the retina (p = 0.2438), (p) but is significantly increased in the RPE/choroid (*p = 0.0436) of JR5558 mice. Data analysed by two-tailed unpaired t test.

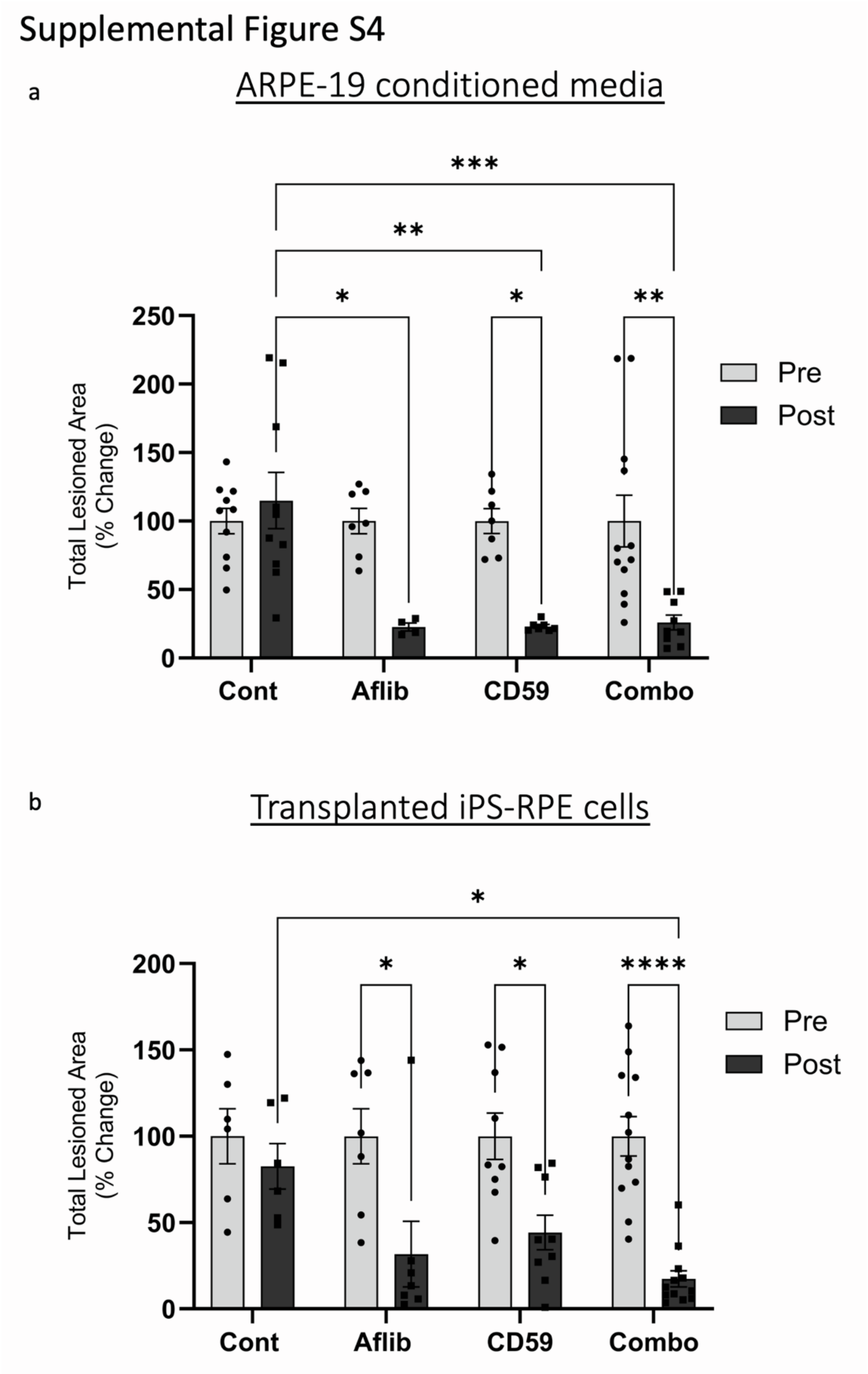

Figure S4. Comparison of relative treatment response following conditioned media or enhanced iPS-RPE injection in JR5558 mice.

a) Comparison of the relative effect of intravitreal injection of control (Cont), aflibercept (Aflib), CD59 and combination (Combo) conditioned media from transfected ARPE-19 cells.

CD59 (*p = 0.0235, n = 7 eyes post injection) and combination (**p = 0.0043, n = 9 eyes post injection) conditioned media significantly reduced lesion area by approximately 70-80% compared to pre-injection fundus fluorescein angiography (FFA) images A similar, non-significant decrease was noted for aflibercept conditioned media (p = 0.0834, n = 4 eyes post injection) **b)** Comparison of the relative effect of subretinal injection of control (Cont, non-transduced), aflibercept (Aflib), CD59, and combination (Combo) enhanced iPS-RPE cells. Aflibercept expressing cells reduced lesion area by approximately 68% (*p = 0.0176, n = 7 eyes), CD59 expressing cells by approximately 56% (*p = 0.0361, n = 9 eyes), and cells expressing both proteins (Combo) by approximately 83% (**p < 0.0001, n = 12 eyes), compared to pre-treatment FFA images. Data analysed by ordinary two-way ANOVA with Tukey's multiple comparisons test.
