# Supplementary material for: Enhanced retinal pigment epithelial cells as a delivery vehicle for retinal disease
Source: Mol Ther Methods Clin Dev. 2025 Mar 14;33(2):101450. doi: 10.1016/j.omtm.2025.101450 (PMC11995081; doi:10.1016/j.omtm.2025.101450)
Supplement: Document S2. Article plus supplemental information [file mmc2.pdf]

Molecular Therapy
Methods & Clinical Development
Original Article

Enhanced retinal pigment epithelial cells as a delivery vehicle for retinal disease

Avril Reddy,[1] Chris Greene,[1] Yosuke Hashimoto,[1] Anna-Sophia Kiang,[1] Natalie Hudson,[1] Peter Adamson,[2] Tiago Santos-Ferreira,[3] and Matthew Campbell[1]

[1]Smurfit Institute of Genetics, Trinity College Dublin, Dublin, Ireland; [2]UCL, Institute of Ophthalmology, University College London, London, UK; [3]Tenpoint Therapeutics, Switzerland Innovation Park, Hegenheimermattweg 167A, 4123 Allschwil, Switzerland

Age-related macular degeneration (AMD) represents a major global health burden, with current estimates suggesting that up to 200 million people are affected globally. While effective treatments exist for the exudative form of the disease termed choroidal neovascular AMD, there remain challenges associated with long-term responses to treatment and the ongoing parallel development of the non-exudative form of AMD. Here, we sought to develop an approach for long-term delivery of both aflibercept, a decoy receptor that neutralises vascular endothelial growth factor and a concomitant treatment focused on treating the non-exudative form of AMD. To this end, we developed a series of induced pluripotent stem cell (iPS)-derived retinal pigment epithelial (RPE) cell lines that stably expressed aflibercept and/or sCD59. These cell lines were shown to produce high concentrations of both proteins. Sub-retinal injection of enhanced RPE cells potently prevented leakage of neovascular lesions in the JR5558 mouse model of retinal and choroidal neovascularization. Early results described here suggest that enhanced iPS-derived RPE cells could represent a novel approach to the long-term delivery of therapeutic agents to the eye.

INTRODUCTION

Age-related macular degeneration (AMD) is the leading cause of blindness in aged populations worldwide.[1,2] The use of intravitreal vascular endothelial growth factor (VEGF)-neutralizing agents has revolutionized the treatment of the neovascular component of AMD referred to as choroidal neovascularization (CNV). However, it is not known to impact other underlying elements of AMD, which also contribute to disease burden and visual acuity (VA) loss. Particular clinical features of the underlying non-vascular forms of AMD, such as thick or thin retinae, thicker sub-retinal tissue, atrophy, or the presence of fibrotic scarring, are all associated with worse baseline VA[3] in clinical trials of VEGF inhibitors. Yet, despite the immediate beneficial effects of VEGF-neutralizing agents, longer-term follow-up of patients has suggested that the vision-saving impact of this medicine is reduced with increasing time on therapy.

It has been reported that the average VA at 5 years with continual VEGF inhibitors declines from year 2 to a level that is below the baseline value at clinical trial entry.[4] Other studies have also demonstrated a decline in VA with continuous anti-VEGF therapy.[5–8] In addition, a significant number of patients treated with anti-VEGF therapy continue to progress to macular atrophy, which then drives the overall downward trend in acuity.[9] Studies have suggested a potential relationship between anti-VEGF therapy and macular atrophy development.[5,6,10–16] Added to this, other studies have suggested that the increase in macular atrophy is a natural consequence of advancing non-exudative AMD.[17,18] Whether VEGF-neutralizing therapies are driving the core elements of non-exudative AMD or it is simply a natural underlying progression, there is a clear need to simultaneously treat both the exudative and non-exudative elements of AMD to optimize clinical outcomes.

Individuals with AMD are also at risk of progressing to the end stage of the non-exudative form of this disease, termed geographic atrophy (GA),[19,20] and will not benefit from anti-VEGF treatment. Until recently, this patient cohort has been advised to modify their diet and make lifestyle changes such as smoking cessation with the aim of slowing disease progression.[21] It is, however, well documented from genetic studies and screening of postmortem donor eye tissues, that complement deposition on ocular tissues is a major contributor to AMD.[22,23]

Variants in the genes that encode complement proteins such as complement factor H, factor I, and C3 have been found in individuals with AMD.[24–26] Additionally, complement proteins have been found in the drusen deposits of AMD affected eyes.[22] While mechanistic studies have gone some way to clarify how these variants affect gene expression and protein function, it is expected that these variants somehow prevent overactivation of the complement pathway.[27] For this reason, methods aimed at regulating complement in AMD have come to the fore in recent years.

There have been numerous clinical trials using novel investigational products that function as complement system inhibitors.[28] In early

Received 19 August 2024; accepted 11 March 2025;
https://doi.org/10.1016/j.omtm.2025.101450.

Correspondence: Matthew Campbell, Smurfit Institute of Genetics, Trinity College Dublin, Dublin, Ireland.
E-mail: matthew.campbell@tcd.ie

© 2025 The Author(s). Published by Elsevier Inc. on behalf of The American Society of Gene and Cell Therapy.
This is an open access article under the CC BY license (http://creativecommons.org/licenses/by/4.0/).

2023, intravitreal pegcetacoplan (Syfovre, Apellis Pharmaceuticals, Inc), a complement C3 inhibitor became the first ever drug approved by the U.S. Food and Drug Administration for the treatment of GA.[29] This was quickly followed by the approval of the C5 inhibitor, avacincaptad pegol (Izervay, Astellas Pharma Inc). In a clinical trial, monthly intravitreal injection of pegcetacoplan significantly slowed GA lesion growth by 21% as compared with non-treated control eyes after 1 year in one trial (OAKS [A Study to Compare the Efficacy and Safety of Intravitreal APL-2 Therapy With Sham Injections in Patients With Geographic Atrophy (GA) Secondary to Age-Related Macular Degeneration]), but failed to reached statistical significance in the other (DERBY [Study to Compare the Efficacy and Safety of Intravitreal APL-2 Therapy With Sham Injections in Patients With Geographic Atrophy (GA) Secondary to Age-Related Macular Degeneration]).[30] A similar result of a 14%–27% reduction in growth was observed after 1 year of monthly injections of avacincaptad pegol.[31,32] Although these results are encouraging, a range of side effects including the transition to wet AMD in 10.14% of eyes treated monthly with pegcetacoplan (untreated was 3.40%) was observed so longer-term monitoring of these individuals will be needed.[33] The transition to neovascular AMD was lower in the case of avacincaptad pegol, with 5% of treated eyes being affected as compared with 3% of sham treated eyes in the GATHER2 (A Phase 3 Safety and Efficacy Study of Intravitreal Administration of Zimura), phase 3 clinical trial.[32]

Compelling programs targeted at complement inhibition are currently under investigation using a diversity of modalities, including gene therapy-based approaches. One program uses adeno-associated virus (AAV)-mediated therapy to express a soluble version of CD59, the endogenous inhibitor of membrane attack complex (MAC) formation.[34,35] Membrane-bound CD59 is normally found at the plasma membrane. where it prevents the recruitment of complement C9 proteins to the C5b-8 complex needed to form a MAC pore in the membrane.[36,37] Normally, overactivation of the complement system results in cell death due to the accumulation of MAC deposits and resulting lysis of the cell.[38] In the initial clinical trial, this gene therapy termed HMR59 seemed to be safe in 17 patients examined, with mild inflammation noted in 4 patients.[39–41] It is currently in phase 2 testing and is also being trialed in combination with anti-VEGF.[42]

While targeting the complement pathway in tandem with VEGF holds immense promise to treat AMD there are delivery as well as regulatory hurdles associated with this approach. Added to this, in many patients, the process of retinal pigment epithelial (RPE) cell atrophy will already have started and, therefore, regulating disease progression as well as focusing on regeneration of damaged tissue will be important considerations. In this regard, we sought to develop and test an optimized and enhanced RPE cell type that could be injected sub-retinally and serve as both a biofactory for therapeutics as well as being able to act as an RPE cell transplant for atrophic AMD eyes. In this way, the enhanced RPE transplant may support its own survival. We sought to modify and enhance these RPE cells such that they would stably express aflibercept (Eylea) in tandem with sCD59. In effect, a single injection of these cells could offer an option for the prolonged expression of therapeutic agents in the diseased eye. Added to this, as techniques evolve for delivery of RPE patches/cell suspensions for transplantation, enhanced RPE cells could offer a highly robust method for treating all stages of AMD with a single injection.

RESULTS

Modified ARPE-19 cells express and secrete aflibercept and sCD59

ARPE-19 cells were transfected with aflibercept and/or soluble human CD59 (sCD59)-containing plasmids. After 48 h, the cell culture supernatant was harvested and screened for expression of secreted proteins, and cells were harvested to examine transcript expression of plasmid DNA (Figure 1). Transfection with increasing concentrations of the aflibercept plasmid alone (single) resulted in a dose-dependent increase in aflibercept protein and transcript expression (Figures 1A and 1C). A similar result was observed following single transfections of the sCD59-expressing plasmid (Figures 1B and 1D). When cells were transfected with both plasmids (combination), both proteins were successfully expressed and secreted into culture media; however, this expression was limited, likely as a result of transfection with high concentrations of plasmid DNA. In combination transfections, expression of aflibercept protein but not transcript, was lower in samples transfected with 400 ng versus 200 ng of each plasmid (Figures 1A–1C). At the transcript level, expression of aflibercept was significantly reduced in cells transfected with 400 ng of both plasmids as compared with 400 ng of the aflibercept plasmid alone (****$p < 0.0001$). Similarly, transfection with 400 ng of both plasmids led to reduced protein and transcript expression of sCD59 compared with transfection with the sCD59 plasmid alone (Figures 1B and 1D, ****$p < 0.0001$ for both comparisons). Interestingly, a low concentration of CD59 was found to be expressed by non-transfected ARPE-19 cells (Figure 1B). Transfection with 400 ng of one or both plasmids was chosen to be the ongoing method to produce conditioned media. This was chosen as it resulted in adequate expression and secretion of aflibercept and sCD59 into culture media following single and combination transfections.

Aflibercept-conditioned media prevents VEGF mediated endothelial cell permeability

Aflibercept works by acting as a decoy receptor neutralizing excess VEGF that would otherwise influence vascular permeability and neovascularization.[43] Here, the effect of a range of VEGF concentrations incubated with control conditioned media or conditioned media from ARPE-19 cells transfected with 400 ng of aflibercept-expressing plasmid for 48 h was examined in an endothelial cell permeability assay. After 24 h, addition of 10 ng/mL VEGF was ineffective, but increased concentrations of VEGF such as 50 ng/mL (****$p < 0.0001$) or 100 ng/mL (****$p < 0.0001$) VEGF to control conditioned media in the basolateral chamber of transwells containing confluent mouse brain endothelial cells resulted in a significant decrease in trans endothelial electrical resistance (TEER), as compared with control conditioned media alone (Figure 2B). At the

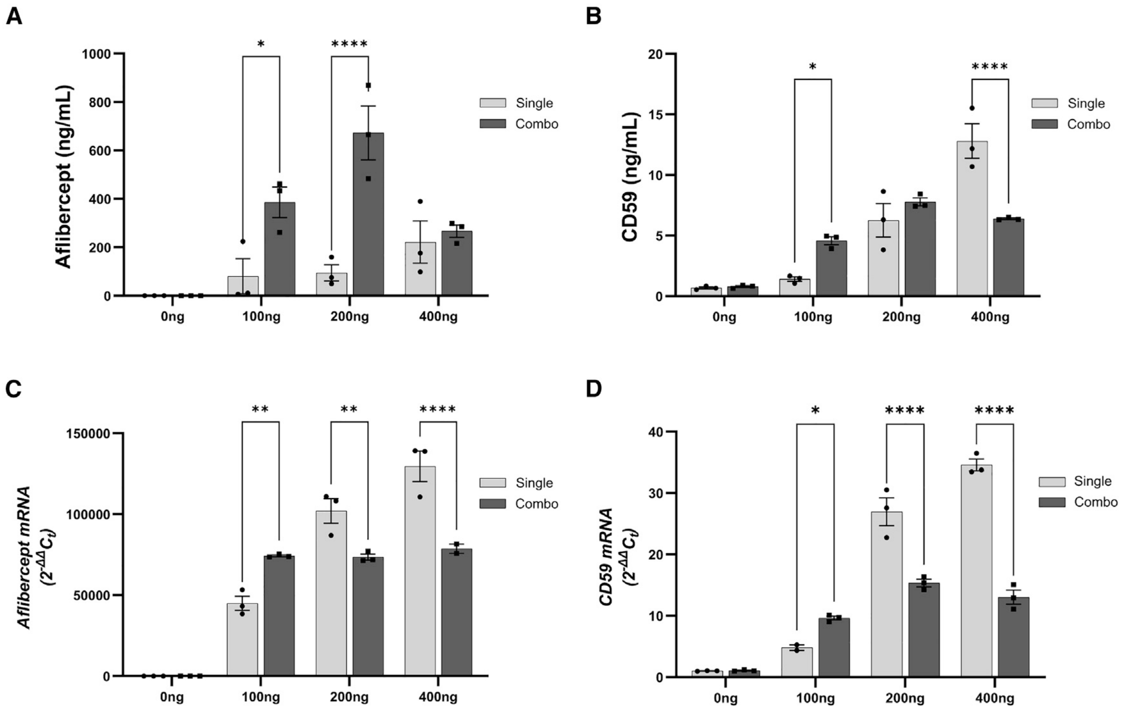

Figure 1. Transient transfection of ARPE-19 cells with aflibercept and sCD59 plasmids results in expression and secretion of both proteins

(A) Conditioned media from ARPE-19 cells transfected with 0, 100, 200, or 400 ng of aflibercept and (B) soluble CD59 (sCD59) expressing plasmid DNA for 48 h contained increasing concentrations of aflibercept or sCD59 protein. Notably, combination transfection (Combo) with 400 ng of both plasmids resulted in decreased expression of sCD59, compared with cells transfected with the sCD59 expressing plasmid alone (single). Additionally, low levels of CD59 expression were noted in non-transfected cells. (C) Transcript analysis shows that after 48 h, transfection with 400 ng of both plasmids (Combo) resulted in reduced expression of aflibercept and (D) CD59 mRNA as compared with transfection with a single plasmid. Data were analyzed by two-way ANOVA with Šidák's multiple comparisons test and graphed using GraphPad Prism 9.0 software. In all comparisons, $n = 3$ independent experiments, error bars represent SEM. * $p < 0.05$; ** $p < 0.01$; **** $p < 0.0001$**.

higher concentration of 100 ng/mL VEGF, a significant increase in fluorescein isothiocyanate (FITC)-dextran 70 kDa tracer flux was observed as compared with control conditioned media (* $p = 0.0286$) (Figure 2C).

Addition of aflibercept-conditioned media (aflibercept concentration = 609.39 ng/mL) mitigated these VEGF-associated permeability changes, with no difference observed at any VEGF concentration compared with control or aflibercept conditioned media without VEGF (Figures 2B and 2C), showing strong suppression of the action of exogenously added VEGF.

sCD59 and combination conditioned media prevents VEGF-induced permeability

This experiment was repeated to examine whether sCD59 or combination conditioned media can also prevent VEGF associated increases in endothelial permeability. Here, aflibercept-conditioned media again prevented the VEGF-induced decreases in TEER that was seen when control conditioned media was used, suggesting that aflibercept sequesters excess VEGF in the media (Figure 2D). Aflibercept-conditioned media (aflibercept concentration = 902.5 ng/mL) alone also caused an increase in TEER, indicating that aflibercept may interact with endogenous VEGF, increasing barrier integrity (**** $p < 0.0001$). The addition of combination conditioned media (aflibercept concentration = 533.69 ng/mL, sCD59 concentration = 15.47 ng/mL) did not cause any significant increases in TEER as compared with control-conditioned media, but again, when 100 ng/mL VEGF was added to the media, no changes in TEER were noted relative to conditioned media alone (**** $p < 0.0001$). Interestingly, sCD59-conditioned media (sCD59 concentration = 20.35 ng/mL) also seemed to mitigate VEGF-mediated decreases in TEER (**** $p < 0.0001$).

sCD59 in conditioned media reduces MAC formation

Conditioned media was added to rodent photoreceptor cell line, 661w on coverslips in 24 well plates and incubated for 72 h. Normal human serum (NHS), a source of complement, or heat-inactivated NHS (HI-NHS), which cannot generate a MAC, was then added to the conditioned media (15% v/v) for 15 min.

The addition of NHS was shown to effectively cause MAC formation, as indicated by C5b9 expression, as compared with cells incubated with HI-NHS (*** $p = 0.001$) (Figures 3A and 3B). Conditioned media containing aflibercept did not significantly reduce complement

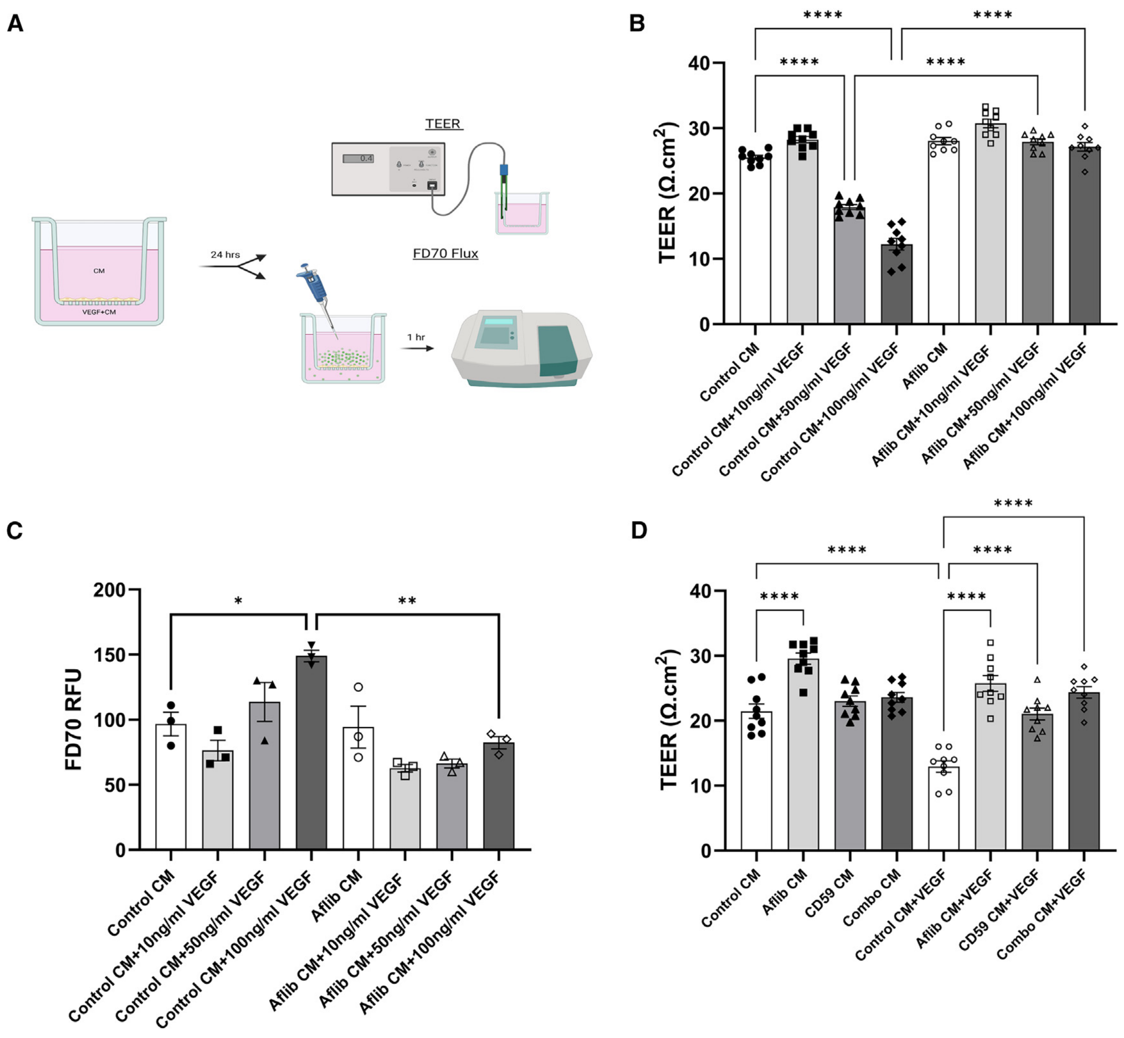

Figure 2. Conditioned media confers protection from VEGF induced permeability

(A) Mouse brain endothelial cells, bEnd.3, were grown to confluence on cell culture inserts with a 0.4-μm pore size. Conditioned media was added to both chambers. VEGF was added to the basolateral chamber of half of the wells. After 24 h, measures of endothelial cell permeability, TEER or FITC-dextran 70kDa (FD70) tracer flux were obtained. (B) After 24 h, addition of 50 ng/mL or 100 ng/mL VEGF to control conditioned media significantly reduced TEER ($\Omega.cm^2$) as compared with conditioned media alone. Incubation of VEGF in aflibercept conditioned media (aflibercept concentration = 609.39 ng/mL) results in significantly higher TEER values relative to control conditioned media at VEGF concentrations of 50 ng/mL and 100 ng/mL n = 9 measurements from three independent experiments. (C) In control conditioned media, 100 ng/mL VEGF causes a significant increase in FD70 tracer flux as compared with samples without VEGF (*p = 0.0286). Again, aflibercept conditioned media prevents increases in endothelial permeability after incubation with 100 ng/mL VEGF (**p = 0.003). RFU, relative fluorescence units. n = 3 independent experiments. (D) Aflibercept conditioned media (aflibercept concentration = 902.5 ng/mL) alone caused increased TEER. Addition of conditioned media containing sCD59 (CD59 concentration = 20.35 ng/mL) or sCD59 and aflibercept (Combo, aflibercept concentration = 533.69 ng/mL, CD59 concentration = 15.47 ng/mL) also prevented VEGF mediated reduction in TEER as compared with control media.

****p < 0.0001 for all comparisons, n = 9 measurements from three independent experiments. Data analyzed by ordinary one-way ANOVA with Tukey's multiple comparisons test and presented as mean ± SEM.

activation, as compared with control conditioned media, suggesting that aflibercept does not play a role in inhibiting the terminal step of complement activation (Figures 3A and 3B).

When sCD59, a complement inhibitor that prevents the formation of the terminal MAC, was present in conditioned media a significant reduction in C5b9 expression was observed, as compared with incubation with control conditioned media (**p = 0.0093) (Figures 3C and 3D).

Incubation with combination conditioned media reveals a significant reduction in C5b9 expression as compared with control conditioned media but the extent of reduction was less than that of sCD59 conditioned media (Figures 3C–3F). Combination conditioned media

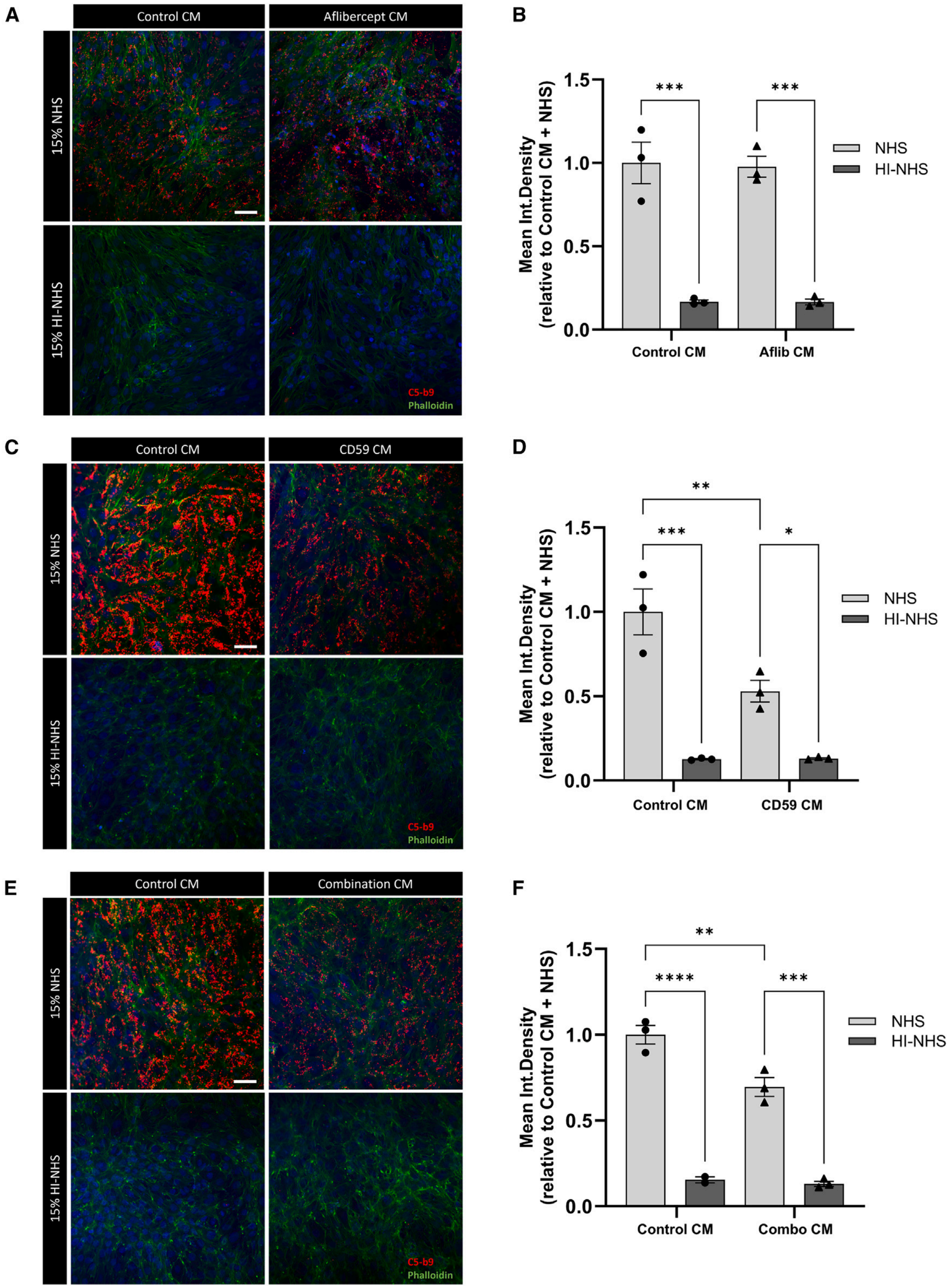

(legend on next page)

contains both aflibercept and sCD59, but the concentration of sCD59 (sCD59 = 7.514 ng/mL) was less than what was present in conditioned media obtained from ARPE-19 cells singly transfected with sCD59 (sCD59 concentration >22.272 ng/mL). This observation implies that CD59 in conditioned media can reduce C5b9 deposition, but there is an expected dose-dependent response for this effect.

Conditioned media produced by enhanced iPS-derived RPE cells prevent endothelial cell permeability

In an effort to move our study to translation to human therapy, we focused on human RPE cells derived from induced pluripotent stem cells (iPS-RPE). These cells were chosen due their greater degree of similarity to human RPE cells and their translatability for clinical applications, such as cell-based therapies. Here, the plasmids used in previous experiments were replaced by AAV2/5 vectors that can express the same sCD59 or aflibercept protein once transduced into the iPS-RPE cells.

Examination of conditioned media from these cells showed similar levels of protein expression to that observed previously. Conditioned media generated from the transduced iPS-RPE cells was added to 661w cells for 48 h before 15% NHS or HI-NHS was added for 30 min. NHS-induced complement activation and MAC formation occurred in all conditions, whereas no activation was noted in cells exposed to HI-NHS (Figures 4A and 4B). There was no difference in the amount of C5b9 expression after incubation with control or aflibercept-conditioned media (Figures 4A and 4B) (aflibercept concentration = 175.21 ng/mL). sCD59-conditioned media also significantly reduced MAC formation, as compared with control media (sCD59 concentration = 41.62 ng/mL; $^{***}p$ = 0.0004). This was also observed after incubation with combination conditioned media (sCD59 concentration = 31.23 ng/mL; aflibercept concentration = 62.39 ng/mL; $^{*}p$ = 0.0449); however, the extent of this reduction was less than observed in CD59-conditioned media alone, likely due to the lower concentration of sCD59 in the combination media.

The addition of 100 ng/mL VEGF to control conditioned media (no AAV) resulted in a significant increase in vascular permeability ($^{***}p$ = 0.0003) (Figure 4C). This increase was prevented by the addition of aflibercept (aflibercept concentration = 822.40 ng/mL), sCD59-conditioned (sCD59 concentration = 50.3 ng/mL) or combination conditioned (sCD59 concentration = 46.644 ng/mL; aflibercept concentration = 446.76 ng/mL) media. Notably, the addition of aflibercept and sCD59 media alone seemed to prevent increased vascular permeability (Figure 4C).

Intravitreal injection of conditioned media from modified ARPE-19 cells into JR5558 mice reduces neovascular lesions

The JR5558 model of spontaneous choroidal and retinal neovascular lesions was chosen as a model of exudative AMD that may show treatment response to secreted target drug, aflibercept. Additionally, retinal cryosections from 8-week-old JR5558 mice show signs of complement activation including C3 deposition around abnormal blood vessels in the outer retina (Figure S2). Transcript analysis of these eyes demonstrated increased expression of *C1qa* ($^{**}p$ = 0.0014) (Figure S3A), *Cfi* ($^{***}p$ = 0.0003) (Figure S3B), and *Cfh* ($^{**}p$ = 0.0025) (Figure S3C) in the retina and *Cfi* ($^{***}p$ = 0.0002) (Figure S3J) in the RPE/choroid as compared with age-matched C57BL/6J mice. Added to this, increased expression of *Vegfa* ($^{**}p$ = 0.0082) (Figure S3G) in the retina and its receptor *Kdr* ($^{*}p$ = 0.0436) (Figure S3P) in the RPE/choroid of these mice confirm their utility for this study examining the therapeutic effect of a VEGF neutralizing agent and a complement inhibitor.

Fundus fluorescein angiography (FFA) images were obtained from JR5558 mice prior to investigation to determine the extent of spontaneous neovascular lesions in each mouse. On day 4 post screening, mice received an intravitreal injection of conditioned media from modified ARPE-19 cells and a final FFA was obtained after 24 h (Figure 5A).

Control conditioned media was found to cause no significant difference in the total area covered by CNV lesions (Figure 5B) (p = 0.9705; n = 10 eyes post injection). In mice that received the aflibercept-conditioned media (aflibercept concentration = 558.97 ng/mL), a significant reduction in the total lesion area was observed after 24 h (Figure 5C) ($^{**}p$ = 0.0095; n = 4 eyes post injection).

Eyes injected with sCD59 conditioned media (sCD59 concentration = 36.81 ng/mL) also demonstrated significant lesion resolution 24 h post injection (Figure 5D) ($^{***}p$ = 0.0006; n = 7 eyes post injection). Interestingly, mice injected with combination conditioned media from cells transfected with the aflibercept and sCD59 expressing plasmids also exhibited a significant resolution of neovascular lesions 24 h post intravitreal injection (Figure 5E) (aflibercept concentration = 259.56 ng/mL; sCD59 concentration = 7.72 ng/mL; $^{**}p$ = 0.0007; n = 9 eyes post injection).

Subretinal injection of iPS-RPE cells in JR5558 mice resolves neovascular lesions

In an effort to translate the enhanced RPE cell-based approach to a translational therapeutic modality, modified iPS-derived human

Figure 3. sCD59 reduces MAC formation in 661w cells exposed to NHS

(A and B) We incubated 661w cells, a rodent photoreceptor cell line, in control or aflibercept (aflibercept concentration = 634.11 ng/mL) conditioned media (CM). After 72 h, 15% v/v NHS or HI-NHS was added to CM for 15 min. MAC formation was visualized by immunocytochemistry for C5-b9 (red) and showed no difference between cells incubated with control or aflibercept CM. Heat-inactivated serum did not cause C5-b9 deposition in either case. (C and D) Incubation with CD59 CM (CD59 concentration >22.27 ng/mL) significantly reduced C5-b9 deposition as compared with control CM following NHS incubation ($^{**}p$ = 0.0093). (E and F) NHS incubation in CM containing both aflibercept and CD59 (aflibercept concentration = 358.18 ng/mL, CD59 concentration = 7.514 ng/mL) again reduced MAC deposition on 661w cells exposed to NHS ($^{**}p$ = 0.0059).

Data analyzed by ordinary one-way ANOVA with Tukey's multiple comparisons test, n = 3 independent experiments, error bars represent SEM. Nuclei are marked by Hoechst 33258. Scale bar, 50 μm.

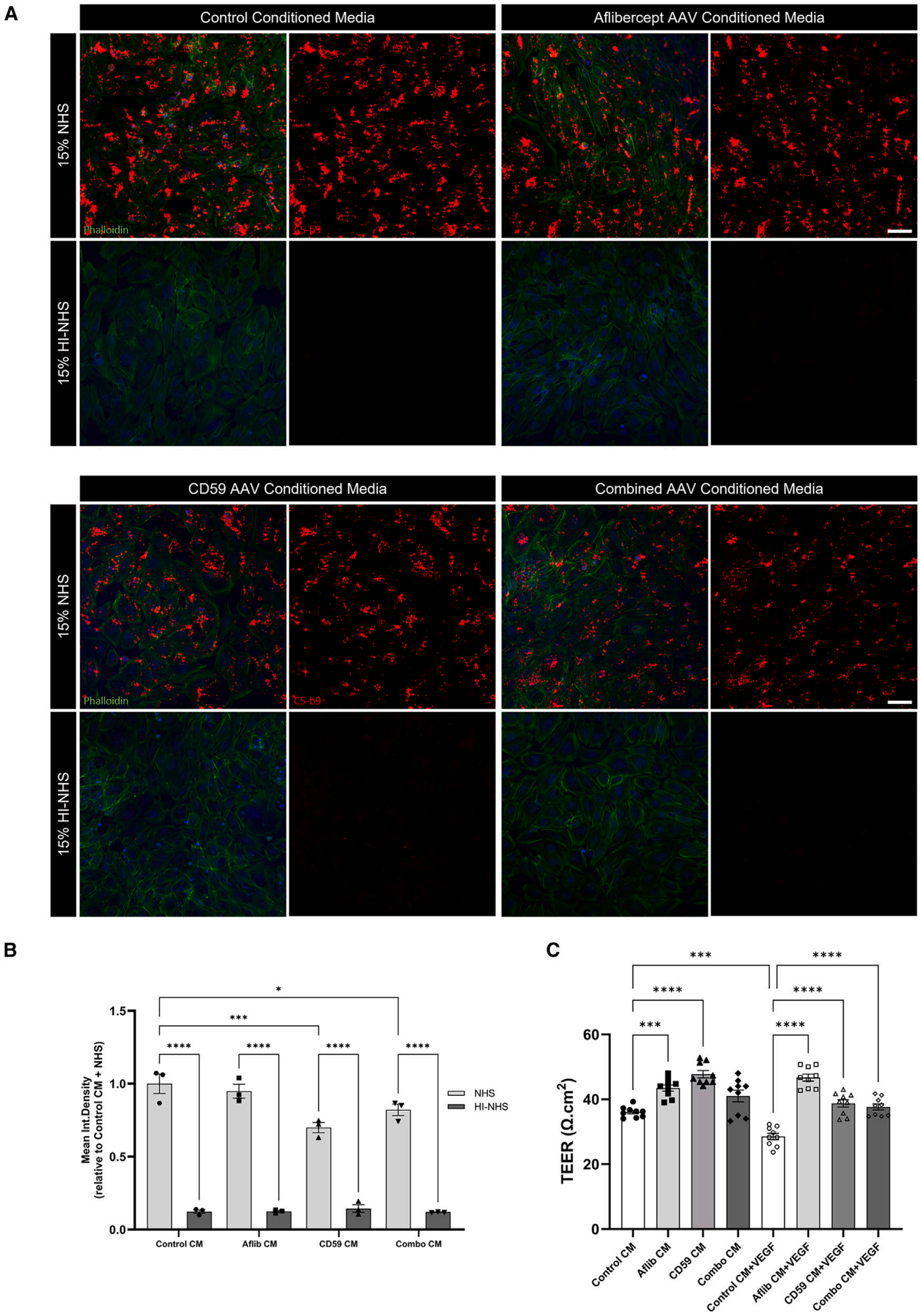

(legend on next page)

RPE cells expressing aflibercept and sCD59 cells were injected subretinally in the peripheral margin of the retina of JR5558 mice (Figure 6A). These mice were imaged by FFA prior to injection, 2 days post injection and 1 week post injection. After 7 days, no significant decrease in the total lesion area was noted in mice injected with control, non-transduced cells (Figure 6B) ($p = 0.2188$; $n = 6$ eyes). However, mice injected with modified cells expressing aflibercept showed a significant resolution of neovascular lesions (Figure 6C) (*p = 0.0312; $n = 7$ eyes; approximately 691.2 cells secreting 1.74 ng of aflibercept every 48 h). In eyes injected with cells expressing only sCD59, a similar result was noted (Figure 6D) (*p = 0.0195; $n = 9$ eyes; approximately 472.5 cells in 1.5 μL suspension secreting 0.063 ng of CD59 every 48 h).

Eyes injected with cells expressing both CD59 and aflibercept (Figure 6E) ($n = 12$ eyes; approximately 360 cells in 1.5 μL suspension secreting 1.069 ng of aflibercept and 0.053ng of CD59 every 48 h) showed the most significant reduction in total lesion area (***p = 0.0005) as compared with images taken before injection. Therefore, a combination-based approach of aflibercept and sCD59 may prove more beneficial than simply aflibercept expression alone.

DISCUSSION

Progress in the field of neovascular AMD has focused intensely on increasing the dose and dosing interval of VEGF-neutralizing agents. However, in the non-exudative space, progress is currently on a new generation of intravitreal-dosed complement inhibitors. Importantly, however, there is a continuing failure to recognize AMD as a single disease. Patients with the non-vascular form of AMD represent the vast majority of cases worldwide. Yet, upon conversion to the exudative form of disease, a significant proportion of patients present with complications defined by the neovascular elements of the underlying AMD. It is now clear that AMD has a number of pathological mechanisms at play simultaneously, and there is a need to treat the mechanisms driving both the exudative and atrophic forms of the disease. Consequently, there are obvious benefits in using a combined treatment approach to limit the impact of multiple mechanisms driving the various aspects of AMD.

Complement inhibitors have been shown to be effective in limiting the expansion of macular atrophy, while a range of other complement targets are in development such as complement factor B and sCD59. There is also evidence from clinical studies that, although VEGF may not itself directly impact the underlying non-exudative AMD processes, there is a consequential increase in complement activation products in the eye following dosing of intravitreal VEGF-neutralizing agents in patients with choroidal neovascularization. These changes in complement activation products are observed a month after two monthly anti-VEGF doses and reduced aqueous humor VEGF levels correlate specifically with the level of complement activation factors in aqueous humor. This strongly suggests a VEGF-neutralizing agent-induced dysregulation of the local complement system.[44] This provides an even greater compulsion to treat both ocular VEGF and ocular complement activation together, using a targeted and sustained pharmacological approach.

While anti-VEGF monotherapy has revolutionized how neovascular eye disease is managed and treated, it is now becoming apparent that major challenges remain in the maintenance of longer-term improvements in patients' vision. Indeed, the recent approval of faricimab, a dual VEGF and Ang-2 inhibitor, is driving the way for additional mechanisms beyond VEGF, but the reality remains that regular intraocular injections are a burden. A shifted focus has also sought to develop long-lasting approaches to delivering therapies to the eye for AMD, increasing and ensuring patient compliance. These include devices such as the port delivery system (Susvimo) developed by Roche as well as gene therapy-based methods aimed at long-term and persistent expression of anti-VEGF agents such as aflibercept.[45,46] Conversely, several studies identified that long-term treatment with anti-VEGF may lead to focal atrophy of RPE cells and, consequently, secondary loss of photoreceptors.[8,9,47]

Here, we sought to develop an enhanced RPE cell that would potentially fulfill three distinct functions. Two functions would be an iPS-derived RPE cell that would produce both aflibercept and sCD59, two potent regulators of distinct mechanisms that are core to the pathology of both exudative and non-exudative AMD, including vascular permeability. A third function, by virtue of the fact that AMD involves damage to the RPE and consequent loss, is the concept that these cells could serve as a source of replacement RPE cells and, in the context of transplantation, could replace central RPE cells lost in GA-AMD and in doing so support their own survival.

In vitro results presented here suggest that inhibiting the terminal step of the complement cascade using sCD59 may provide a way to

Figure 4. CM from iPS-RPE cells is protective

(A and B) Conditioned media (CM) was collected from sCD59 AAV2/5 and/or aflibercept AAV2/5 vector transduced iPS-RPE cells every 48 h. CM was added to confluent 661w cells for 48 h and then incubated with 15% v/v NHS or HI-NHS for 30 min. Again, aflibercept CM (aflibercept concentration = 175.21 ng/mL) did not provide any protection against MAC formation as compared with control (no AAV) CM. CD59 (CD59 concentration = 41.62 ng/mL) and combination (CD59 concentration = 31.23 ng/mL, aflibercept concentration = 62.39 ng/mL) CM did reduce C5-b9 deposition (red) as compared with control CM (***p = 0.0004 and *p = 0.0449 respectively) (scale bar, 50 μm). Nuclei are marked by Hoechst 33258. $n = 3$ independent experiments. (C) After 24 h, aflibercept (aflibercept concentration = 822.40 ng/mL) and CD59 (CD59 concentration = 50.3 ng/mL) CM added to bEnd.3 cells in tissue culture inserts increased TEER (Ω.cm^2, ***p = 0.0005, ****$p < 0.0001$ respectively) relative to control CM. Addition of 100 ng/mL VEGF to control CM in the basolateral chamber decreased TEER (***p = 0.0003). When added to aflibercept, CD59 or combination (CD59 concentration = 46.644 ng/mL, aflibercept concentration = 446.76 ng/mL) CM, VEGF was prevented from causing increases in endothelial permeability (****$p < 0.0001$ for all comparisons). Data analyzed by ordinary one-way ANOVA with Tukey's multiple comparisons test, $n = 9$ measurements from three independent experiments, represented as mean ± SEM.

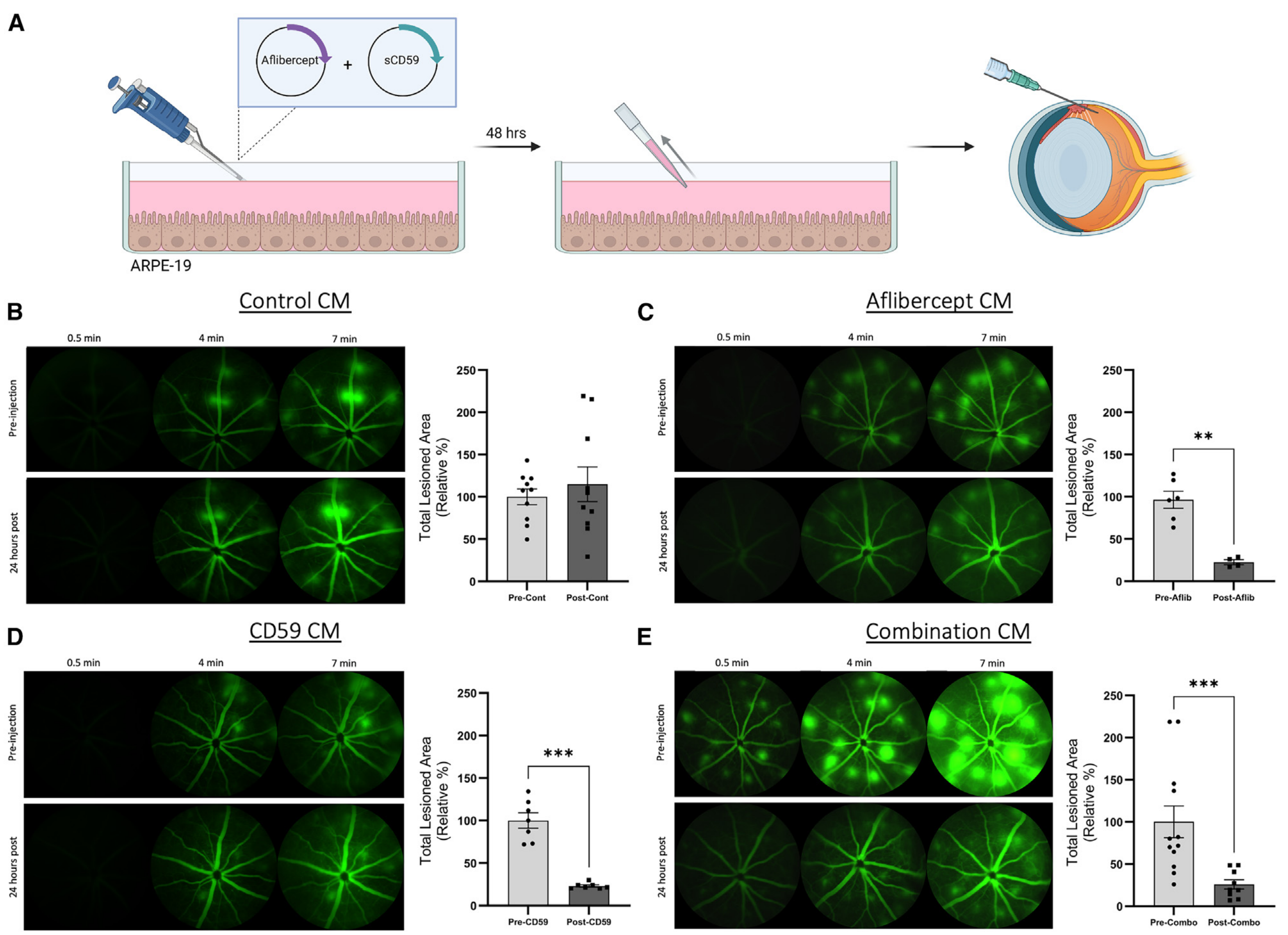

Figure 5. CM from modified ARPE-19 cells reduces neovascular lesions in the JR5558 mouse
(A) ARPE-19 cells were transiently transfected with the aflibercept, sCD59, or both plasmids and conditioned media (CM) was harvested after 48 h. This CM was injected intravitreal into pre-screened JR5558 mice. (B) Intravitreal injection of control CM from ARPE-19 cells did not result in any changes to the total area covered by CNV-like lesions after 24 h, when compared with FFA images taken before injection (p = 0.9705; n = 10 eyes post injection). (C) Aflibercept CM (aflibercept concentration = 558.97 ng/mL) significantly reduced lesion area 24 h after intravitreal injection (**p = 0.0095; n = 4 eyes post injection). (D) Similarly, intravitreal injection of CD59 CM (CD59 concentration = 36.81 ng/mL) resulted in a significant reduction in the total area covered by CNV lesions (***p = 0.0006; n = 7 eyes post injection). (E) Combination CM (aflibercept concentration = 259.56 ng/mL; CD59 concentration = 7.72 ng/mL) produced similar results (***p = 0.0007; n = 9 eyes post injection).
Data analyzed by Mann-Whitney test or unpaired two-tailed t test and presented as the percentage change, relative to lesion area pre-injection with error bars indicating SEM.

directly limit the lytic activity of MAC formation and deposition that is known to cause cell death in both the dry and wet forms of AMD. Added to this, sCD59 seems to be very potent at inhibiting retinal vascular permeability *in vivo* in the JR5558 model and *in vitro* in neural endothelial cells, yet the underlying mechanism that drives this effect is yet to be elucidated. Although the use of a soluble form of CD59 has become popular in neovascular AMD research, its effect at limiting lesion area has previously been attributed to its role in reducing MAC formation and endothelial cell lysis.[34,48] Interestingly, endothelial CD59 expression has previously been shown to increase in hypoxic conditions, following statin administration, or following experimental sheer stress.[49–51] In these cases, upregulated expression was described to be a response to inflammation; however, results presented in this manuscript suggest that further research is needed to elucidate whether CD59 expression may have a broader therapeutic role in AMD.

When delivered in combination with an anti-VEGF therapeutic such as aflibercept, we envision this approach could extend the therapeutic window and prevent periods of low drug exposure due to bolus dosing for both classes of therapeutic, which will likely result in improved clinical outcomes. Early results presented here suggest that it will be possible to inject cells in the margins of the retina, thereby having an option to limit any effect on VA due to cell placement or place them centrally in cases of GA. In effect, the injected cells would act as a biofactory for the production of aflibercept and sCD59. In this study, iPS-RPE cells modified *ex vivo* seemed to produce and secrete therapeutic proteins into the vitreous *in vivo*,

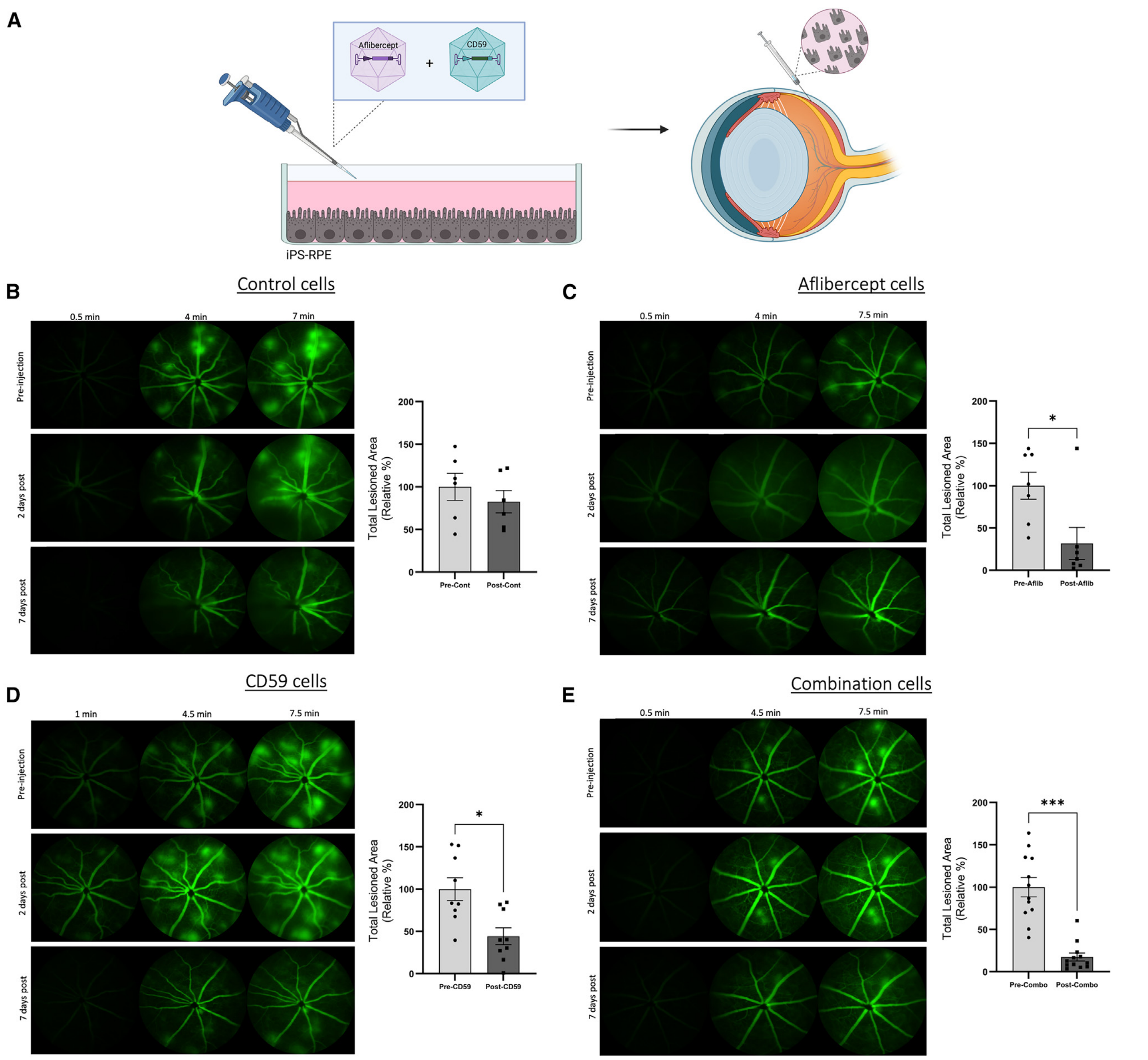

Figure 6. Subretinal injection with enhanced RPE cells leads attenuates neovascular lesions in JR5558 mice

(A) Human iPS-RPE cells were transduced with an aflibercept and/or sCD59 expressing AAV2/5 vector. After a few weeks, cells are detached and delivered via subretinal injection under the margin of the peripheral neuroretina. (B) JR5558 mice were imaged by FFA prior to injection with non-transduced iPS-RPE cells (control) and then 2 and 7 days post injection. At 7 days post injection, there is no significant decrease in the total area covered by CNV-like lesions ($p = 0.2188$; $n = 6$ eyes; approximately 691.2 cells in 1.5 μL suspension). (C) Subretinal injection with aflibercept AAV2/5-transduced iPS-RPE cells significantly decreases total lesion area after 7 days (*$p = 0.0312$; $n = 7$ eyes; approximately 691.2 cells secreting 1.74 ng of aflibercept every 48 h). (D) iPS-RPE cells expressing sCD59 also led to a significant reduction in CNV-like lesion area 7 days post injection (*$p = 0.0195$; $n = 9$ eyes, approximately 472.5 cells in 1.5 μL suspension secreting 0.063 ng of sCD59 every 48 h). (E) Subretinal injection of cells transduced with both AAVs dramatically reduced lesion area following subretinal cell injection (***$p = 0.0005$; $n = 12$ eyes, approximately 360 cells in 1.5 μL suspension secreting 1.069 ng of aflibercept and 0.053 ng of sCD59 every 48 h).

Data analyzed by Wilcoxon, paired two-tailed t test and presented as the percentage change, relative to lesion area pre-injection with error bars indicating SEM.

following subretinal delivery. Additionally, combination treatment resulted in an equal or greater resolution of lesions compared with the delivery of either recombinant protein alone (Figure S4).

Poor patient compliance is undoubtedly an ongoing issue for the successful treatment of AMD. Clinical trial data that led to the approval of the anti-VEGF drugs, including aflibercept and

ranibizumab, or the complement inhibitors, pegcetacoplan and avacincaptad pegol, reported on the therapeutic benefit following monthly or every other month dosing. Real-world data have now shown that for anti-VEGF treatment, up to 57% of patients discontinue treatment within 12 months or irregularly return to the clinic.[52,53] As this will likely also be an issue for the treatment of GA with approved complement inhibitors, the need to reduce the required number of clinical visits is apparent.

This technique is admittedly in its infancy, but we speculate that improvements in cell transplantation techniques could be applied using these enhanced RPE cells in the future. In this regard, a larger study is warranted to examine the long-term survival and expression stability of transplanted enhanced RPE cells. As discussed above, the consequence of the continual production of therapeutic proteins within the retina remains to be fully elucidated. Recent data from a phase 1 clinical trial on 30 patients with neovascular AMD suggest that the continual local production of aflibercept following intravitreal delivery of ADVM-022 (ixoberogene soroparvovec, ixo-vec, Adverum Biotechnologies) is relatively safe for up to 2 years.[54] We would highlight that, if an issue with the safety of continual overdosing should arise, current gene therapy approaches (such as ixo-vec) leave a permanent genetic signature in transduced retinal cells. Although an inducible therapeutic approach may be necessary, for now, transplanted enhanced RPE cells may be a safer or more conservative approach for extended local production of therapeutic molecules as we envision that these cells will not remain indefinitely within the retina. Targeting AMD holistically using our modified RPE-based approach has the potential to change the landscape of how this disease is managed clinically, with the potential for improved clinical outcomes.

MATERIALS AND METHODS

Confirmation of plasmid sequences

Plasmid aliquots, aflibercept pcDNA3.1 and shCD59 pcDNA3.1, were obtained from Tenpoint Therapeutics. A double restriction digest using the enzymes EcoRI (BioLabs, #R010L), BamHI-HF (BioLabs, #R3136L), and XbaI, (BioLabs, #R0145L) released a single fragment from the plasmid backbone and was visualized on a 1% agarose gel. We then used 10 ng of the plasmid to transform chemically competent *E. coli* DH5α cells as outlined by the manufacturer's guidelines (Thermo Fisher Scientific catalog #EC0111) and plated out on LB plates supplemented with 100 mg/mL ampicillin. A single ampicillin-resistant transformant colony was picked and cultured overnight in selective medium to provide the inoculum for a large-scale maxiprep, which was carried out using a Qiagen Hi-Speed Plasmid Maxi Kit (ref. 12663) and the manufacturer's instructions. We sent 100 ng subsample of this to Eurofins Genomics for DNA sequence determination using standard pcDNA3.1 forward and reverse primers. The sequence was translated *in silico* and sequence identity was confirmed to match with published sequences (Aflibercept sequence, Drugbank: DB08885 and *Homo sapiens* CD59, transcript variant 1, NM_203330.2) (Figure S1).

ARPE-19 cell maintenance and generation of conditioned media

ARPE-19 cells were maintained in DMEM/F12 (1:1 mixture of DMEM and Ham's F12, Gibco, 11320033) supplemented with 10% heat-inactivated fetal bovine serum (HI-FBS) (Sigma-Aldrich, F7524). (FBS was heat inactivated at 56°C for 30 min.) All cells were incubated at 37°C under 5% CO_2. ARPE-19 cells were detached using Trypsin-EDTA (Gibco, T4049).

For transfections, cells were in a 24-well plate at a density of 2×10^5 cells/mL (100,000 cells per well). After 24 h, media was replaced with serum free DMEM/F12 media and sub-confluent cells were transfected with 100 ng, 200 ng, or 400 ng of the plasmid of interest (aflibercept, shCD59 or both) using lipofectamine 3000 reagents (Invitrogen, L3000008) and reduced serum media, Opti-MEM (Gibco, 31985047). Media was harvested after 48 h, centrifuged at 1,000 rpm for 5 min to remove any floating cells, and aliquoted for storage at −70°C. The concentration of secreted aflibercept (AssayGenie, HUMB00054) or CD59 (Abcam, ab263893) protein was measured by ELISA and interpolated using a standard curve (four-parameter logistic non-linear regression) with GraphPad Prism 9.0 software.

RNA was isolated from transfected cells using the Total RNA Kit lysis buffer and the E.Z.N.A Total RNA kit (Omega Bio-Tek, R6834-02) according to manufacturer's guidelines and stored at −70°C. cDNA was prepared using High Capacity cDNA Reverse Transcription Kit (Applied Biosystems, 4368814) using 10 μL of 10 ng/μL RNA and the resultant cDNA was diluted 1:10 in nuclease-free water before use in qPCR analysis. qPCR was performed on a StepOnePlus (Applied Biosystems) machine using SensiFAST SYBR Hi-ROX (Bioline, BIO-92020) and primers designed using known plasmid sequences; *sCD59* forward, 5′- CTGACTGCAAAACAGCCGTC-3′; reverse, 5′-TTTTCCCTCAAGCGGGTTGT-3′; aflibercept forward, 5′- CAGAGT GACAAGCCCCAACA-3′; reverse, 5′- GTCAGCAGGCCGATCTC TTT-3′; and the housekeeping gene, *β-Actin* forward, 5′- GAGCACA GAGCCTCGCCTTT-3′; reverse, 5′- TCATCATCCATGGTGAGC TGG-3′. PCR conditions with added melt-curve analysis are as follows: 95°C 20 s; (95°C 3 s, 60°C 30 s) ×40; 95°C 15 s, 60°C for 1 min, 0.3°C increments with data collection to 95°C, 95°C 15 s. Data were analyzed using the comparative delta delta cycle threshold (C_t) method and normalized to the housekeeping gene, *β-Actin*, to generate the delta C_t for each well. Results were analyzed by two-way ANOVA with Šidák's multiple comparisons test and graphed using GraphPad Prism 9.0 software.

Samples transfected with 400 ng of plasmid DNA were chosen to be used in future experiments because of the adequate protein expression, from single and combination transfections, and secretion into the conditioned media. Each aliquot of conditioned media was only thawed once to prevent any harmful effects of repeated freeze-thaw cycles.

Complement deposition assays

We maintained 661w cells, a rodent photoreceptor cell line, in DMEM containing 4,500 mg/L glucose, GlutaMAX and

110.0 mg/mL sodium pyruvate (DMEM, Gibco, 31966-021) supplemented with 1% v/v HI-FBS (Sigma). Cells were passaged as above. We placed 13-mm coverslips (Sarstedt, 83.1840.002) in a 24 well-plate and coated with fibronectin (1:20 in PBS, Sigma, F1141) for 1 h at 37°C, 5% CO_2, and then washed with Dulbecco's PBS (14190169). Cells were passaged as above and seeded on coverslips at 2×10^5 cells/mL (105,000 cells per well). After 24 h, media was removed and replaced with conditioned media obtained from transfected ARPE-19 cells.

After 72 h, 15% v/v NHS (Sigma, S1764) or HI-NHS was added to the conditioned media in the wells and incubated for 15 min. Media was removed and cells were washed in PBS and then fixed in 4% w/v paraformaldehyde (PFA) for 10 min at room temperature (RT). Fixed cells were blocked in 5% v/v normal goat serum (NGS, Sigma, G9023) in PBS with 0.05% Triton X-100 (PBST) and incubated with monoclonal mouse anti-C5b9 (aE11, 1:75, Santa Cruz Biotechnology, sc-58935) and Alexa Fluor 488-conjugated phalloidin (1:100, Invitrogen, A12379) in 1% NGS-PBST, overnight at 4°C. Cells were washed three times in PBS and incubated in goat anti-mouse Cy3 (1:500, Invitrogen, A10521) in 1% NGS in PBST for 2 h at RT. Cells were washed three more times and counterstained with Hoechst 33258 (1:10,0000, Sigma, B22261) for 3 min. Cells were washed a final three times and then coverslips were mounted on slides using Aqua Poly/Mount (Polysciences, 18606-5). Coverslips were imaged using a Zeiss LSM 710 confocal laser scanning microscope. The levels of C5b9 staining were quantified using ImageJ (Fiji) software and an ordinary one-way ANOVA with Tukey's multiple comparisons test was performed using GraphPad Prism 9.0 Software. Data presented as mean ± standard error of the mean (SEM).

Permeability assays

Mouse brain endothelial cells (bEnd.3, ATCC, CRL-2299), maintained in DMEM supplemented with 10% v/v FBS, were passaged as above. Polyethylene therephthalate TC inserts, 0.3 cm^2 growth area, 0.4-μm pore size (Sarstedt, 83.3932.041), were placed in a 24-well plate and coated with fibronectin (1:20 in PBS) for 1 h at 37°C and washed once with PBS. We seeded 5×10^4 cells into each insert with growth media added to the apical and basolateral chambers. After 72 h, or when cells were deemed confluent, media in the basolateral chamber was replaced with conditioned media from ARPE-19 transfection experiments with or without recombinant mouse VEGF 164 (R&D Systems, 493-MV-025). After 24 h, measurements of TEER were obtained using the EVOM2 epithelial voltohmmeter with chopstick electrodes. The long arm of the probe was placed in the basolateral chamber and the short arm in the apical chamber. TEER values were recorded in triplicate measurements and expressed as ohm*cm^2 ($\Omega.cm^2$).

At this time point, 200 μL of tracer (1 mg/mL FITC-dextran 70 kDa [Sigma, FD70]) in DMEM culture media) was added to the apical chamber and the plate was returned to 37°C and 5% CO_2. After 1 h, a sample of media was removed from the basolateral chamber and transferred to a 96-well flat bottom plate. A spectrofluorometer was used to determine tracer fluorescence in this media at an excitation wavelength of 485 nm and an emission wavelength of 520 nm. These relative fluorescent units and TEER values were graphed and analyzed using an ordinary one-way ANOVA with Tukey's multiple comparisons test and presented as mean ± SEM on GraphPad Prism 9.0 software.

JR5558 animal experiments

All animal experiments were performed in accordance with the ARVO statement for the Use of Animals in Ophthalmic and Vision Research and the European Communities Council Directive (2010/63/EU). Mice were housed under a 12-h light/dark cycle with temperature of 18°C–23°C and humidity of 40%–50% maintained, and provided *ad libitum* access to standard food and water. Prior to ocular imaging or injection, mice were anaesthetized by subcutaneous injection of 100 mg/kg ketamine and 0.25 mg/kg medetomidine hydrochloride (Sedastart, Animalcard Ltd.) and pupils were dilated using 1% tropicamide and 2.5% phenylephrine eye drops. Anesthesia was reversed using 1 mg/kg atipamezole hydrochloride (Sedastop, Animalcare Ltd.). JR5558 mice, a model with spontaneous choroidal neovascular lesions were used to determine the efficacy of conditioned media, *in vivo*. Eight-week-old mice were pre-screened by FFA to determine the relative burden of choroidal neovascular lesions before treatment. Both eyes were imaged and treated as individual values. After 96 h, eyes were intravitreally injected with conditioned media from ARPE-19 cells transfected with the relevant plasmids. In these transfection experiments, DMEM/F12 was replaced with a serum-free media, X-Vivo 15 (Lonza, LZBE02-060F) that was less likely to cause off-target immune responses in the eye. After 24 h, additional FFA images were taken as a post-injection time point. The relative total lesion area, determined by the amount of fluorescein leakage in areas of choroidal neovascular lesions, was compared before and after injection using pixel intensity measurements. Results were analyzed using GraphPad Prism 10.4 by the Mann-Whitney test or unpaired two-tailed t test and presented as the percentage change, relative to lesion area before injection.

Eight-week-old JR5558 mice were sacrificed by cervical dislocation and eyes were fixed in 4% PFA for 90 min RT. After fixation, the lens was removed, eyecups were cryopreserved using an increasing gradient of sucrose solution (concentrations of 10%, 20%, or 30%), diluted in PBS, and then embedded in optimal cutting temperature (OCT, VWR, 361603E) and frozen in liquid nitrogen. Retinal cryosections of 12 μm thickness were collected using a Leica Biosystems CM1950 cryostat and transferred to poly-L-lysine coated slides and stored at −20°C.

Sections were permeabilized in PBST for 30 min RT and blocked in 5% NGS in PBST for 45 min and then washed three times with PBS. Slides were incubated with mouse rabbit anti-C3 (1:100, Abcam, ab11887) and isolectin-IB_4-Alexa Fluor 568 (1:300, Invitrogen, I21412) primary antibodies diluted in 1% NGS in PBST, overnight at 4°C. Slides were washed three more times before incubation with goat anti-rabbit 488 (1:500, Invitrogen, A11008) diluted in 1% NGS

Table 1. Primer sequences used in qPCR analyses

Primer target	Direction	Sequence 5′-3′
β-Actin (mouse)	forward	GGGAAATCGTGCGTGACAT
	reverse	GTGATGACCTGGCCGTCAG
C1qa (mouse)	forward	GTGGCTGAAGATGTCTGCCGAG
	reverse	TTAAAACCTCGGATACCAGTCCG
Cfi (mouse)	forward	GAGCCGTTGTGAAGACCGA
	reverse	TCCGATCACTCGTTTCCTGC
Cfh (mouse)	forward	GAGACAAGCAGGAGTACGAACG
	reverse	CCATCCAAGTATTTCACGGTGGT
C3 (mouse)	forward	TTCCTTCACTATGGGACCAGC
	reverse	CTCCAGCCGTAGGACATTGG
C3ar1 (mouse)	forward	TGCTCAGCAACTCGTCCAAT
	reverse	ACTCCATGGCTCAGTCAAGC
C5 (mouse)	forward	GGACAAAACTTGGGGACAGGA
	reverse	TACACGTGAGAGACTGGGCT
C7 (mouse)	forward	ACTGTGGGGGAGACAAGAGC
	reverse	GGCCATAGGAGTCCCACTTG
Vegfa (mouse)	forward	TATTCAGCGGACTCACCAGC
	reverse	AACCAACCTCCTCAAACCGT
Kdr (mouse)	forward	TCCACATGGGCGAATCACTC
	reverse	GAGTGTGCCAGCCTACTACA

βactin was used as housekeeping gene for normalization of relative gene expression.

PBST for 2 h, RT. Slides were washed three more times and counterstained with Hoechst 33258 (1:10,0000) for 3 min. Slides were washed a final three times and coverslips were mounted using Aqua Poly/Mount (Polysciences) and left to dry overnight. Images were captured using a Zeiss LSM 710 confocal laser scanning microscope and processed using Zeiss Zen 2012 software.

Eight-week-old JR5558 and C57BL/6J mice were sacrificed, and eyes were dissected to isolate the neuroretina and the RPE/choroid for RNA extraction and qPCR analysis as above (primers in Table 1). Data were analyzed using an unpaired t test for each gene, comparing JR5558 with C57BL/6J retinas or RPE/choroid and graphed as mean ± SEM using GraphPad Prism 9.0.

iPS-RPE cells maintenance and AAV transduction

Human iPS-RPE cells were obtained from Fujifilm Cellular Dynamics (R1101). Cells were defrosted rapidly and added to 10 mL of freshly prepared culture media (Table 2). We coated 24-well plates with 2.5 μg/mL vitronectin (StemCell, 7180) in CellAdhere Dilution Buffer (StemCell, 7183) for 1 h RT and then washed them in the dilution buffer. Cells were centrifuged at 1,000 rpm for 5 min, the supernatant was discarded, and the cell pellet was resuspended in 1 mL of fresh media. A cell count was performed, and cells were seeded at 0.3×10^6 cells/mL (180,000 cells per well) into the coated wells of the 24-well plate. Culture media was replenished every 48 h. After 7 days, cells were infected with an AAV2/5 capable of expressing aflibercept or sCD59 protein. AAV infection occurred in minimal growth media (200 μL) at a multiplicity of infection of 1e5 (10,000), given that each well contained 300,000 cells. For combination AAV infections, total MOI was 2e5 (half from each AAV vector). After 9 h, 400 μL culture media was added and left overnight. Virus containing media was replaced with fresh culture media the following morning. After 24 h, media was again replaced with fresh culture media and subsequently collected every 48 h from this point and considered to be conditioned media.

Table 2. Components and volumes required to prepare 100mL of culture media for iPS-RPE cell maintenance

Component	Volume (mL)	Final concentration
MEM alpha (Thermo Fisher Scientific,1271063)	93.3	91.3%
Knockout SR (Thermo Fisher Scientific, 10828028)	5	5%
N-2 supplement (Thermo Fisher Scientific, 17502048)	1	1%
Hydrocortisone, 50 μM (Sigma, H6909)	0.11	55nM
Taurine, 50 mg/mL (Sigma, T0625)	0.5	250 μg/mL
Triiodo-L-thyronine (Sigma, T5516) 20 μg/mL, diluted 1:1,000 before use	0.07	14 pg/mL
Gentamicin, 50 mg/mL (Thermo Fisher Scientific, 15750060)	0.05	25 μg/mL

Once prepared, media was sterile filtered using a 0.2-μm PES filter and stored for a maximum of 2 weeks at 4°C.

Subretinal injection of iPS-RPE in JR5558 mice

To test the efficacy of a cell transfer technique *in vivo*, iPS-RPE cells were delivered via subretinal injection into the peripheral region of the retina in 13-week-old JR5558 mice. These cells were previously transduced with the relevant AAV as above. After a few weeks, cell culture media was removed and StemPro accutase (Gibco, A1110501) was added to the cells for 15 min (37°C, 5% CO_2). Cells were manually detached by pipetting up and down a few times and transferred to a centrifuge tube with 5 mL of cell culture media. Cells were centrifuged at 1,000 rpm for 5 min, the supernatant was discarded and cells were resuspended in 50 μL of X-Vivo 15 media. A sample of the cell suspension was mixed with trypan blue, and a cell count was performed. Due to the variable recovery of cells following detachment, the expected production of each target protein was corrected for the number of cells injected in each experiment. The remaining cell suspension was placed on ice and 1.5 μL was rapidly used for subretinal injection into each eye of JR5558 mice. Mice received an FFA 72 h prior to injection (pre-screen), 48 h after injection and 7 days post injection. The relative area covered by neovascular lesions, as determined by regions of fluorescein leakage, was measured and compared pre-injection and 7 days post injection. Results were analyzed using GraphPad Prism 10.4 by Wilcoxon, paired two-tailed t test and presented as the percentage change, relative to lesion area pre-injection, as mean ± SEM.

DATA AVAILABILITY

All data are described in the paper and are available on request.

ACKNOWLEDGMENTS

This work was supported by grants from TenPoint Therapeutics, SFI (Eye-D-21/SPP/3732) to M.C., The Irish Research Council (IRC), and by a research grant from SFI under grant number 21/RC/10294_P2 and co-funded under the European Regional Development fund by FutureNeuro industry partners to M.C. The Campbell lab is also supported by a grant from the European Research Council (ERC – Retina-Rhythm). The lab is also supported by Fighting Blindness Ireland.

AUTHOR CONTRIBUTIONS

A.R., study design, experimentation, analysis, and writing. C.G., Y.H., A.S.K., and N.H., Experimentation. P.A., and T.F., Study design. M.C., study design, supervision, and writing.

DECLARATION OF INTERESTS

This study was funded by TenPoint Therapeutics. TenPoint Therapeutics also own IP associated with enhanced RPE cells.

SUPPLEMENTAL INFORMATION

Supplemental information can be found online at https://doi.org/10.1016/j.omtm.2025.101450.

REFERENCES

1. Wong, W.L., Su, X., Li, X., Cheung, C.M.G., Klein, R., Cheng, C.-Y., and Wong, T.Y. (2014). Global prevalence of age-related macular degeneration and disease burden projection for 2020 and 2040: a systematic review and meta-analysis. Lancet. Glob. Health *2*, e106–e116.

2. Deng, Y., Qiao, L., Du, M., Qu, C., Wan, L., Li, J., and Huang, L. (2022). Age-related macular degeneration: Epidemiology, genetics, pathophysiology, diagnosis, and targeted therapy. Genes Dis. *9*, 62–79.

3. Jaffe, G.J., Martin, D.F., Toth, C.A., Daniel, E., Maguire, M.G., Ying, G.-S., Grunwald, J.E., and Huang, J.; Comparison of Age-related Macular Degeneration Treatments Trials Research Group (2013). Macular morphology and visual acuity in the comparison of age-related macular degeneration treatments trials. Ophthalmology *120*, 1860–1870.

4. Comparison of Age-related Macular Degeneration Treatments Trials CATT Research Group, Maguire, M.G., Martin, D.F., Ying, G.S., Jaffe, G.J., Daniel, E., Grunwald, J.E., Toth, C.A., Ferris, F.L., 3rd, and Fine, S.L. (2016). Five-Year Outcomes with Anti-Vascular Endothelial Growth Factor Treatment of Neovascular Age-Related Macular Degeneration: The Comparison of Age-Related Macular Degeneration Treatments Trials. Ophthalmology *123*, 1751–1761.

5. Gillies, M.C., Campain, A., Barthelmes, D., Simpson, J.M., Arnold, J.J., Guymer, R.H., McAllister, I.L., Essex, R.W., Morlet, N., and Hunyor, A.P.; Fight Retinal Blindness Study Group (2015). Long-Term Outcomes of Treatment of Neovascular Age-Related Macular Degeneration: Data from an Observational Study. Ophthalmology *122*, 1837–1845.

6. Rofagha, S., Bhisitkul, R.B., Boyer, D.S., Sadda, S.R., and Zhang, K.; SEVEN-UP Study Group (2013). Seven-year outcomes in ranibizumab-treated patients in ANCHOR, MARINA, and HORIZON: a multicenter cohort study (SEVEN-UP). Ophthalmology *120*, 2292–2299.

7. Writing Committee for the UK Age-Related Macular Degeneration EMR Users Group (2014). The neovascular age-related macular degeneration database: multicenter study of 92 976 ranibizumab injections: report 1: visual acuity. Ophthalmology *121*, 1092–1101.

8. Jaffe, G.J., Ying, G.-S., Toth, C.A., Daniel, E., Grunwald, J.E., Martin, D.F., and Maguire, M.G.; Comparison of Age-related Macular Degeneration Treatments Trials Research Group (2019). Macular Morphology and Visual Acuity in Year Five of the Comparison of Age-related Macular Degeneration Treatments Trials. Ophthalmology *126*, 252–260.

9. Gillies, M.C., Hunyor, A.P., Arnold, J.J., Guymer, R.H., Wolf, S., Pecheur, F.L., Munk, M.R., and McAllister, I.L. (2020). Macular Atrophy in Neovascular Age-Related Macular Degeneration: A Randomized Clinical Trial Comparing Ranibizumab and Aflibercept (RIVAL Study). Ophthalmology *127*, 198–210.

10. Comparison of Age-related Macular Degeneration Treatments Trials CATT Research Group, Martin, D.F., Maguire, M.G., Fine, S.L., Ying, G.s., Jaffe, G.J., Grunwald, J.E., Toth, C., Redford, M., and Ferris, F.L., 3rd (2012). Ranibizumab and bevacizumab for treatment of neovascular age-related macular degeneration: two-year results. Ophthalmology *119*, 1388–1398.

11. IVAN Study Investigators, Chakravarthy, U., Harding, S.P., Rogers, C.A., Downes, S.M., Lotery, A.J., Wordsworth, S., and Reeves, B.C. (2012). Ranibizumab versus bevacizumab to treat neovascular age-related macular degeneration: one-year findings from the IVAN randomized trial. Ophthalmology *119*, 1399–1411.

12. Cho, H.J., Yoo, S.G., Kim, H.S., Kim, J.H., Kim, C.G., Lee, T.G., and Kim, J.W. (2015). Risk factors for geographic atrophy after intravitreal ranibizumab injections for retinal angiomatous proliferation. Am. J. Ophthalmol. *159*, 285–292.e1.

13. Chakravarthy, U., Harding, S.P., Rogers, C.A., Downes, S.M., Lotery, A.J., Culliford, L.A., and Reeves, B.C.; IVAN study investigators (2013). Alternative treatments to inhibit VEGF in age-related choroidal neovascularisation: 2-year findings of the IVAN randomised controlled trial. Lancet *382*, 1258–1267.

14. Bailey, C., Scott, L.J., Rogers, C.A., Reeves, B.C., Hamill, B., Peto, T., Chakravarthy, U., and Harding, S.P.; writing committee for the IVAN Study Group (2019). Intralesional Macular Atrophy in Anti-Vascular Endothelial Growth Factor Therapy for Age-Related Macular Degeneration in the IVAN Trial. Ophthalmology *126*, 75–86.

15. Grunwald, J.E., Pistilli, M., Ying, G.-S., Maguire, M.G., Daniel, E., and Martin, D.F.; Comparison of Age-related Macular Degeneration Treatments Trials Research Group (2015). Growth of geographic atrophy in the comparison of age-related macular degeneration treatments trials. Ophthalmology *122*, 809–816.

16. Kuroda, Y., Yamashiro, K., Ooto, S., Tamura, H., Oishi, A., Nakanishi, H., Miyata, M., Hata, M., Takahashi, A., Wakazono, T., et al. (2018). MACULAR ATROPHY AND MACULAR MORPHOLOGY IN AFLIBERCEPT-TREATED NEOVASCULAR AGE-RELATED MACULAR DEGENERATION. Retina *38*, 1743–1750.

17. Wykoff, C.C., Ou, W.C., Brown, D.M., Croft, D.E., Wang, R., Payne, J.F., Clark, W.L., Abdelfattah, N.S., and Sadda, S.R.; TREX-AMD Study Group (2017). Randomized Trial of Treat-and-Extend versus Monthly Dosing for Neovascular Age-Related Macular Degeneration: 2-Year Results of the TREX-AMD Study. Ophthalmol. Retina *1*, 314–321.

18. Mantel, I., Zola, M., De Massougnes, S., Dirani, A., and Bergin, C. (2019). Factors influencing macular atrophy growth rates in neovascular age-related macular degeneration treated with ranibizumab or aflibercept according to an observe-and-plan regimen. Br. J. Ophthalmol. *103*, 900–905.

19. Bakri, S.J., Bektas, M., Sharp, D., Luo, R., Sarda, S.P., and Khan, S. (2023). Geographic atrophy: Mechanism of disease, pathophysiology, and role of the complement system. J. Manag. Care Spec. Pharm. *29*, S2–S11.

20. Keenan, T.D.L. (2023). Geographic Atrophy in Age-Related Macular Degeneration: A Tale of Two Stages. Ophthalmol. Sci. *3*, 100306.

21. Carneiro, Â., and Andrade, J.P. (2017). Nutritional and Lifestyle Interventions for Age-Related Macular Degeneration: A Review. Oxid. Med. Cell. Longev. *2017*, 6469138.

22. Mullins, R.F., Russell, S.R., Anderson, D.H., and Hageman, G.S. (2000). Drusen associated with aging and age-related macular degeneration contain proteins common to extracellular deposits associated with atherosclerosis, elastosis, amyloidosis, and dense deposit disease. FASEB J. *14*, 835–846.

23. Fritsche, L.G., Igl, W., Bailey, J.N.C., Grassmann, F., Sengupta, S., Bragg-Gresham, J.L., Burdon, K.P., Hebbring, S.J., Wen, C., Gorski, M., et al. (2016). A large genome-wide association study of age-related macular degeneration highlights contributions of rare and common variants. Nat. Genet. *48*, 134–143.

24. Haines, J.L., Hauser, M.A., Schmidt, S., Scott, W.K., Olson, L.M., Gallins, P., Spencer, K.L., Kwan, S.Y., Noureddine, M., Gilbert, J.R., et al. (2005). Complement factor H variant increases the risk of age-related macular degeneration. Science *308*, 419–421.

25. Kavanagh, D., Yu, Y., Schramm, E.C., Triebwasser, M., Wagner, E.K., Raychaudhuri, S., Daly, M.J., Atkinson, J.P., and Seddon, J.M. (2015). Rare genetic variants in the CFI gene are associated with advanced age-related macular degeneration and commonly result in reduced serum factor I levels. Hum. Mol. Genet. *24*, 3861–3870.

26. Yates, J.R.W., Sepp, T., Matharu, B.K., Khan, J.C., Thurlby, D.A., Shahid, H., Clayton, D.G., Hayward, C., Morgan, J., Wright, A.F., et al. (2007). Complement C3 variant and the risk of age-related macular degeneration. N. Engl. J. Med. *357*, 553–561.
27. Loyet, K.M., Deforge, L.E., Katschke, K.J., Jr., Diehl, L., Graham, R.R., Pao, L., Sturgeon, L., Lewin-Koh, S.-C., Hollyfield, J.G., and van Lookeren Campagne, M. (2012). Activation of the alternative complement pathway in vitreous is controlled by genetics in age-related macular degeneration. Investig. Ophthalmol. Vis. Sci. *53*, 6628–6637.
28. Qin, S., Dong, N., Yang, M., Wang, J., Feng, X., and Wang, Y. (2021). Complement Inhibitors in Age-Related Macular Degeneration: A Potential Therapeutic Option. J. Immunol. Res. *2021*, 9945725.
29. Csaky, K.G., Miller, J.M.L., Martin, D.F., and Johnson, M.W. (2024). Drug Approval for the Treatment of Geographic Atrophy: How We Got Here and Where We Need to Go. Am. J. Ophthalmol. *263*, 231–239.
30. Heier, J.S., Lad, E.M., Holz, F.G., Rosenfeld, P.J., Guymer, R.H., Boyer, D., Grossi, F., Baumal, C.R., Korobelnik, J.-F., Slakter, J.S., et al. (2023). Pegcetacoplan for the treatment of geographic atrophy secondary to age-related macular degeneration (OAKS and DERBY): two multicentre, randomised, double-masked, sham-controlled, phase 3 trials. Lancet *402*, 1434–1448.
31. Jaffe, G.J., Westby, K., Csaky, K.G., Monés, J., Pearlman, J.A., Patel, S.S., Joondeph, B.C., Randolph, J., Masonson, H., and Rezaei, K.A. (2021). C5 Inhibitor Avacincaptad Pegol for Geographic Atrophy Due to Age-Related Macular Degeneration: A Randomized Pivotal Phase 2/3 Trial. Ophthalmology *128*, 576–586.
32. Khanani, A.M., Patel, S.S., Staurenghi, G., Tadayoni, R., Danzig, C.J., Eichenbaum, D.A., Hsu, J., Wykoff, C.C., Heier, J.S., Lally, D.R., et al. (2023). Efficacy and safety of avacincaptad pegol in patients with geographic atrophy (GATHER2): 12-month results from a randomised, double-masked, phase 3 trial. Lancet *402*, 1449–1458.
33. NCT03525600 (2023). Study to Compare the Efficacy and Safety of Intravitreal APL-2 Therapy With Sham Injections in Patients With Geographic Atrophy (GA) Secondary to Age-Related Macular Degeneration.
34. Cashman, S.M., Ramo, K., and Kumar-Singh, R. (2011). A non membrane-targeted human soluble CD59 attenuates choroidal neovascularization in a model of age related macular degeneration. PLoS One *6*, e19078.
35. Ramo, K., Cashman, S.M., and Kumar-Singh, R. (2008). Evaluation of adenovirus-delivered human CD59 as a potential therapy for AMD in a model of human membrane attack complex formation on murine RPE. Investig. Ophthalmol. Vis. Sci. *49*, 4126–4136.
36. Couves, E.C., Gardner, S., Voisin, T.B., Bickel, J.K., Stansfeld, P.J., Tate, E.W., and Bubeck, D. (2023). Structural basis for membrane attack complex inhibition by CD59. Nat. Commun. *14*, 890.
37. Ninomiya, H., and Sims, P.J. (1992). The human complement regulatory protein CD59 binds to the alpha-chain of C8 and to the "b"domain of C9. J. Biol. Chem. *267*, 13675–13680.
38. Kumar-Singh, R. (2019). The role of complement membrane attack complex in dry and wet AMD - From hypothesis to clinical trials. Exp. Eye Res. *184*, 266–277.
39. NCT03144999 (2021). Treatment of Advanced Dry Age Related Macular Degeneration With AAVCAGsCD59.
40. Khan, H., Aziz, A.A., Sulahria, H., Khan, H., Ahmed, A., Choudhry, N., Narayanan, R., Danzig, C., and Khanani, A.M. (2023). Emerging Treatment Options for Geographic Atrophy (GA) Secondary to Age-Related Macular Degeneration. Clin. Ophthalmol. *17*, 321–327.
41. Lad, E.M., Chao, D.L., Pepio, A., Zhang, W., Capuano, G., Rogers, A., Baker, B.J., Cohen, M., Nida Sen, H., and Heier, J.S. (2023). Pooled safety analysis of a single intravitreal injection of JNJ-1887 (gene therapy, AAVCAGsCD59) in patients with age-related macular degeneration (AMD). Investig. Ophthalmol. Vis. Sci. *64*, 732.
42. NCT03585556 (2022). AAVCAGsCD59 for the Treatment of Wet AMD.
43. Adams, B.S., Sorhaitz, W., and Stringham, J. (2023). Aflibercept (StatPearls Publishing).
44. Tanaka, K., Oguchi, Y., Omori, T., Ishida, Y., Shintake, H., Tomita, R., Kasai, A., Ogasawara, M., Sugano, Y., Itagaki, K., et al. (2021). Changes in complement activation products after anti-VEGF injection for choroidal neovascularization in age-related macular degeneration and pachychoroid disease. Sci. Rep. *11*, 8464.
45. Lanzetta, P., Korobelnik, J.-F., Heier, J.S., Leal, S., Holz, F.G., Clark, W.L., Eichenbaum, D., Iida, T., Xiaodong, S., Berliner, A.J., et al. (2024). Intravitreal aflibercept 8 mg in neovascular age-related macular degeneration (PULSAR): 48-week results from a randomised, double-masked, non-inferiority, phase 3 trial. Lancet *403*, 1141–1152.
46. Campochiaro, P.A., Avery, R., Brown, D.M., Heier, J.S., Ho, A.C., Huddleston, S.M., Jaffe, G.J., Khanani, A.M., Pakola, S., Pieramici, D.J., et al. (2024). Gene therapy for neovascular age-related macular degeneration by subretinal delivery of RGX-314: a phase 1/2a dose-escalation study. Lancet *403*, 1563–1573.
47. Foss, A., Rotsos, T., Empeslidis, T., and Chong, V. (2022). Development of Macular Atrophy in Patients with Wet Age-Related Macular Degeneration Receiving Anti-VEGF Treatment. Ophthalmologica *245*, 204–217.
48. Bai, T., Cui, B., Xing, M., Chen, S., Zhu, Y., Lin, D., Guo, Y., Du, M., Wang, X., Zhou, D., and Yan, H. (2024). Stable inhibition of choroidal neovascularization by adeno-associated virus 2/8-vectored bispecific molecules. Gene Ther. *31*, 511–523.
49. Kinderlerer, A.R., Steinberg, R., Johns, M., Harten, S.K., Lidington, E.A., Haskard, D.O., Maxwell, P.H., and Mason, J.C. (2006). Statin-induced expression of CD59 on vascular endothelium in hypoxia: a potential mechanism for the anti-inflammatory actions of statins in rheumatoid arthritis. Arthritis Res. Ther. *8*, R130.
50. Gelfand, B.D., and Ambati, J. (2016). A revised hemodynamic theory of age-related macular degeneration. Trends Mol. Med. *22*, 656–670.
51. Cui, X., Zhang, X., Bu, H., Liu, N., Li, H., Guan, X., Yan, H., Wang, Y., Zhang, H., Ding, Y., and Cheng, M. (2017). Shear stress-mediated changes in the expression of complement regulatory protein CD59 on human endothelial progenitor cells by ECM-integrinαVβ3-F-actin pathway in vitro. Biochem. Biophys. Res. Commun. *494*, 416–421.
52. Lad, E.M., Hammill, B.G., Qualls, L.G., Wang, F., Cousins, S.W., and Curtis, L.H. (2014). Anti-VEGF treatment patterns for neovascular age-related macular degeneration among Medicare beneficiaries. Am. J. Ophthalmol. *158*, 537–543.e2.
53. Polat, O., İnan, S., Özcan, S., Doğan, M., Küsbeci, T., Yavaş, G.F., and İnan, Ü.Ü. (2017). Factors affecting compliance to intravitreal anti-vascular endothelial growth factor therapy in patients with age-related macular degeneration. Turk. J. Ophthalmol. *47*, 205–210.
54. Khanani, A.M., Boyer, D.S., Wykoff, C.C., Regillo, C.D., Busbee, B.G., Pieramici, D., Danzig, C.J., Joondeph, B.C., Major, J.C., Jr., Turpcu, A., and Kiss, S. (2024). Safety and efficacy of ixoberogene soroparvovec in neovascular age-related macular degeneration in the United States (OPTIC): a prospective, two-year, multicentre phase 1 study. EClinicalMedicine *67*, 102394.

OMTM, Volume *33*

Supplemental information

Enhanced retinal pigment epithelial cells as a delivery vehicle for retinal disease

Avril Reddy, Chris Greene, Yosuke Hashimoto, Anna-Sophia Kiang, Natalie Hudson, Peter Adamson, Tiago Santos-Ferreira, and Matthew Campbell

Supplemental Figures

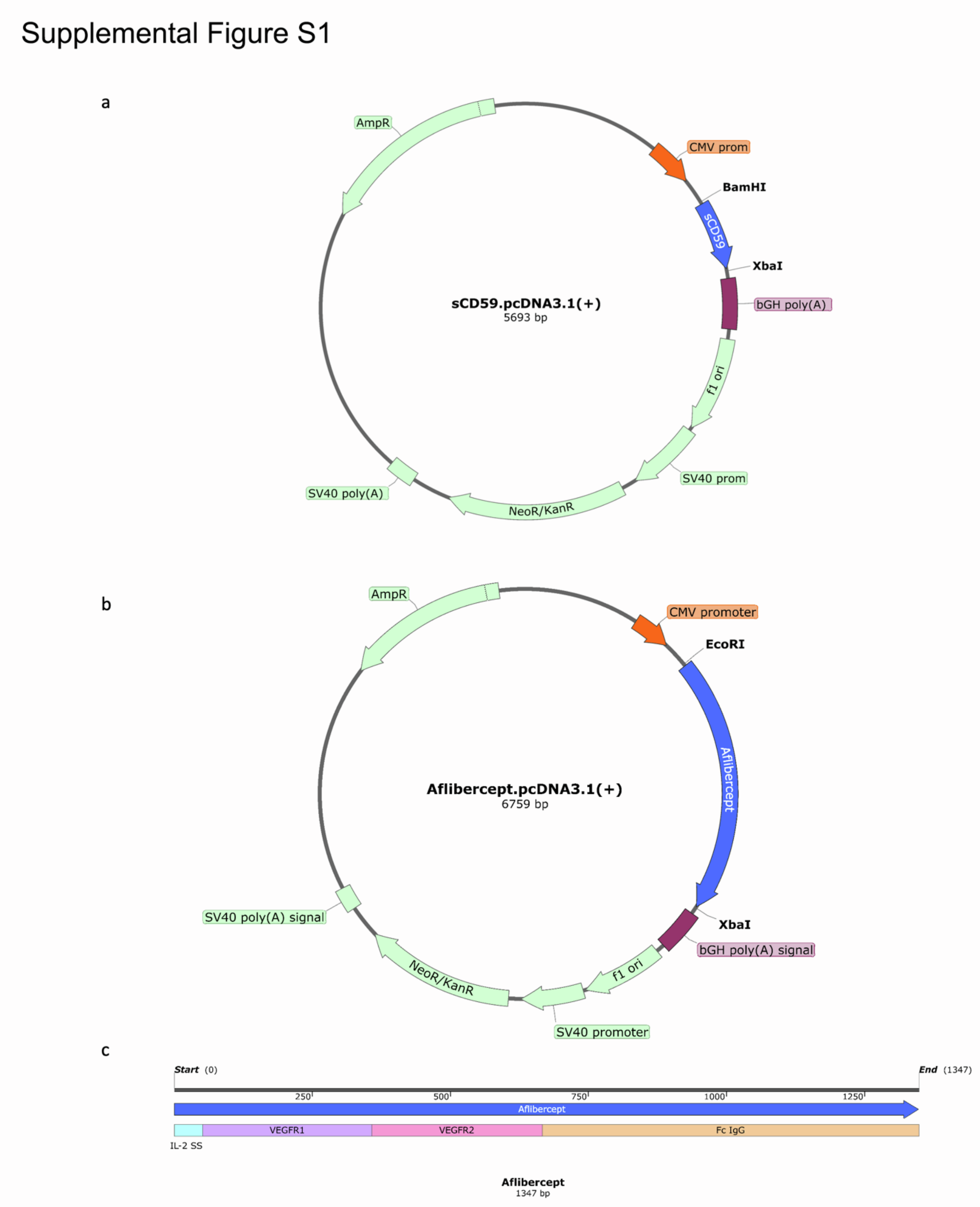

Figure S1. Graphic maps of plasmids used for ARPE-19 transfection.

a) Maps of the sCD59 and **b)** aflibercept expressing plasmids containing the target cDNA (blue) that was confirmed to share sequence identity with published sequences (Homo Sapiens CD59, transcript variant 1, (NM_203330.2) and Drugbank Aflibercept sequence (DB08885),

respectively). Target cDNA is flanked by the BamHI or EcoRI and Xba1 restriction enzyme recognition sites. The ampicillin resistance region (AmpR, green), was utilised for selection of a transformant colony prior to large scale maxiprep. **c)** The aflibercept cDNA sequence contains the interleukein-2 secretion signal peptide (IL-2 SS), vascular endothelial growth factor receptor (VEGFR) 1, VEGFR2 and Fc fragment of human IgG1 immunoglobulin (Fc IgG) sequences.

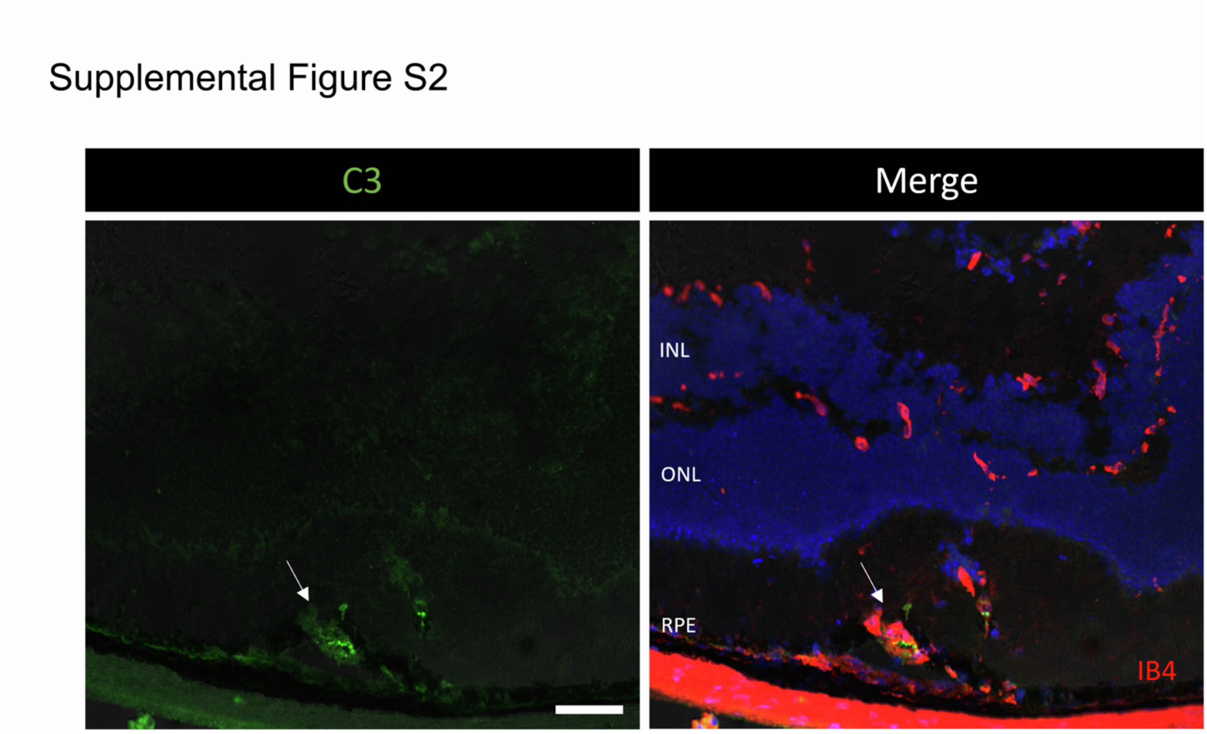

Figure S2. Complement, C3, is observed in areas of neovascular lesions in JR5558 mice. The JR5558 mouse model of choroidal neovascularisation (CNV) shows signs of complement activation such as C3 deposition (green, white arrows) around abnormal blood vessels (red, isolectin-B_4, IB4) in the outer retina. Nuclei marked by Hoechst 33258. INL: inner nuclear layer, ONL: outer nuclear layer, RPE: retinal pigment epithelium. Scale bar = 50µm.

Supplemental Figure S3

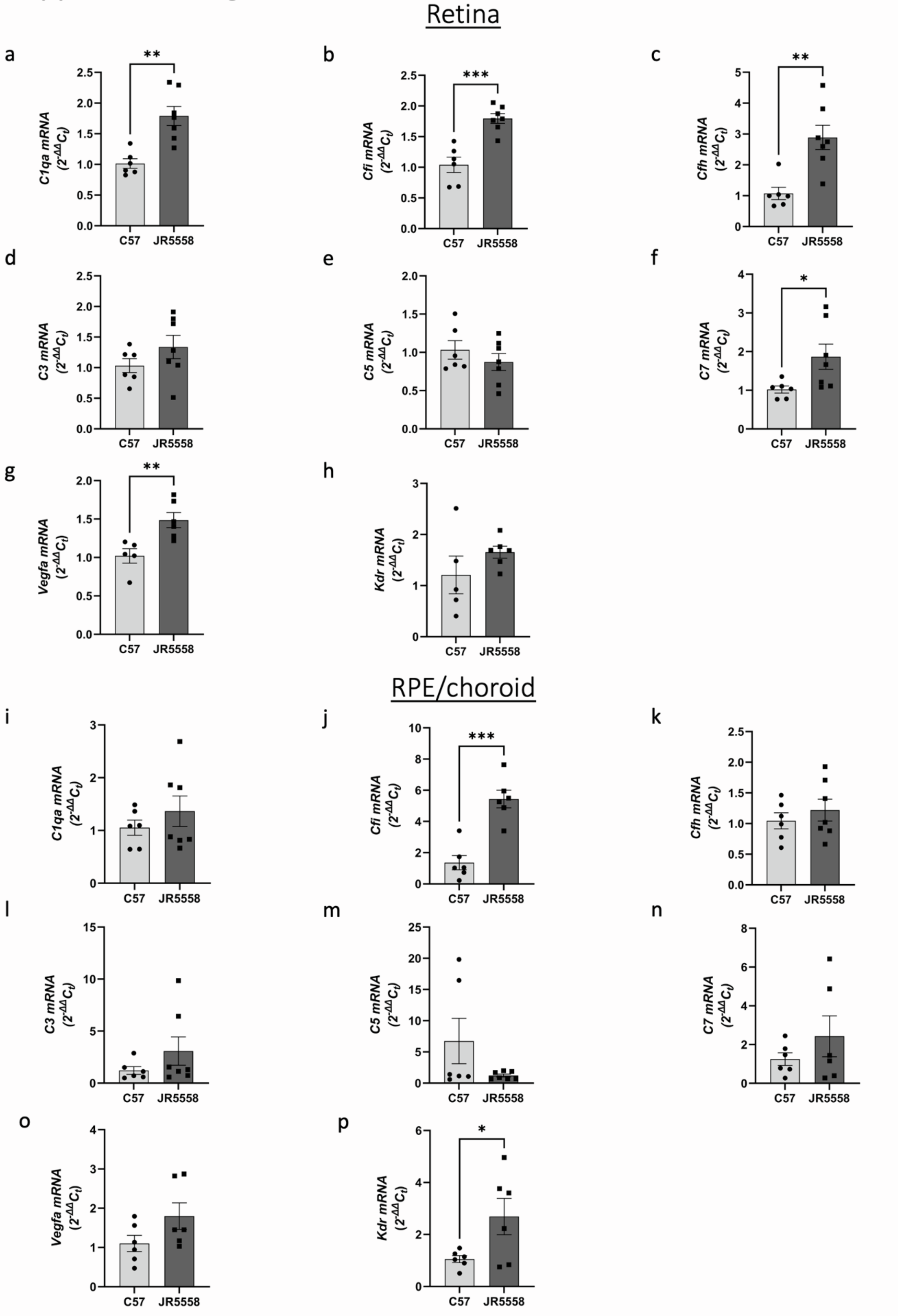

Figure S3. Transcript expression of several complement components and members of the Vegf pathway are upregulated in the retina and RPE/choroid of JR5558 mice.

Signs of complement activation were noted in the retinas of 8-week-old JR5558 mice compared to age-matched C57BL/6J mice. Increased expression of components (a) *C1qa* (**p = 0.0014), (b) *Cfi* (***p = 0.0003), (c) *Cfh* (**p = 0.0025) and (f) *C7* (*p = 0.0401) in the retina and (j) *Cfi* (***p = 0.0002) in the RPE/choroid indicate that the complement system may be activated in this spontaneous neovascular model. Expression of (d) *C3* (p = 0.2142), and (e) *C5* (p = 0.3536) in the retina and (i) *C1qa* (p = 0.3787), (k) *Cfh* (p = 0.4578), (l) *C3* (p = 0.2461), (m) *C5* (p = 0.1452) and (n) *C7* (p = 0.3109) in the RPE/choroid are not significantly different in JR5558 mice. (g) Transcript expression of *Vegfa* is significantly increased in the retina (**p = 0.0082), (o) but not in the RPE/choroid (p = 0.1082) of JR5558 mice. (h) Conversely, *Kdr,* (the murine homolog of VEGFR2) is unchanged in the retina (p = 0.2438), (p) but is significantly increased in the RPE/choroid (*p = 0.0436) of JR5558 mice. Data analysed by two-tailed unpaired t test.

Supplemental Figure S4

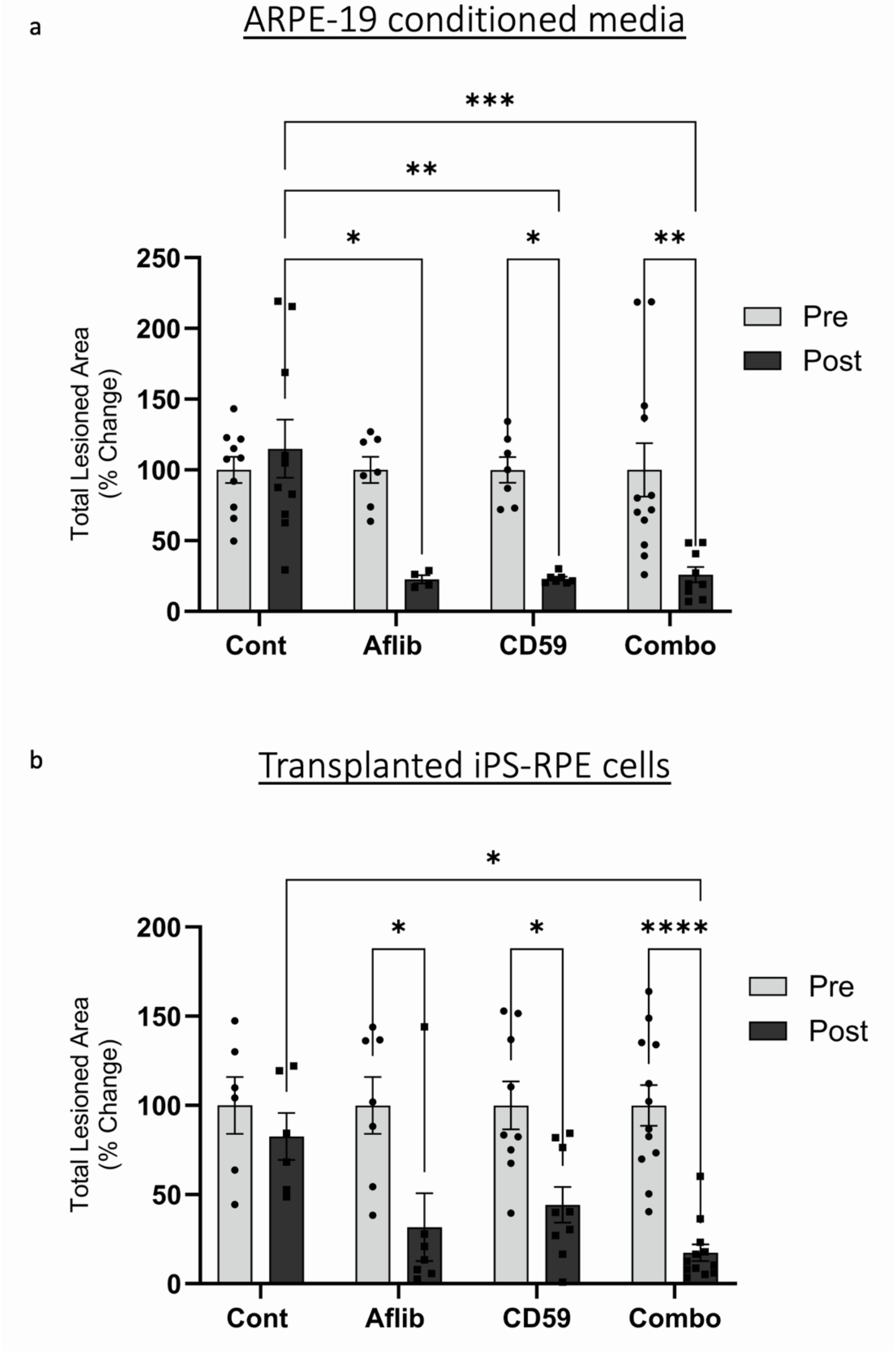

Figure S4. Comparison of relative treatment response following conditioned media or enhanced iPS-RPE injection in JR5558 mice.

a) Comparison of the relative effect of intravitreal injection of control (Cont), aflibercept (Aflib), CD59 and combination (Combo) conditioned media from transfected ARPE-19 cells.

CD59 (*p = 0.0235, n = 7 eyes post injection) and combination (**p = 0.0043, n = 9 eyes post injection) conditioned media significantly reduced lesion area by approximately 70-80% compared to pre-injection fundus fluorescein angiography (FFA) images A similar, non-significant decrease was noted for aflibercept conditioned media (p = 0.0834, n = 4 eyes post injection) **b)** Comparison of the relative effect of subretinal injection of control (Cont, non-transduced), aflibercept (Aflib), CD59, and combination (Combo) enhanced iPS-RPE cells. Aflibercept expressing cells reduced lesion area by approximately 68% (*p = 0.0176, n = 7 eyes), CD59 expressing cells by approximately 56% (*p = 0.0361, n = 9 eyes), and cells expressing both proteins (Combo) by approximately 83% (**p < 0.0001, n = 12 eyes), compared to pre-treatment FFA images. Data analysed by ordinary two-way ANOVA with Tukey's multiple comparisons test.
